# DFRL-Mol: a dual-stage framework of reinforcement learning for multi-scenario molecule optimization

**DOI:** 10.1093/bib/bbag493

**Published:** 2026-09-10

**Authors:** Zhenyi Wu, Pengcheng Zhao, Jianing Li, Qiong Wang, Qin Zhang, Hui Yu, Zhe Yu, Jian-Yu Shi

**Affiliations:** School of Life Science and Technology, Northwestern Polytechnical University, 127 West Youyi Road, Beilin District, Xi’an, Shaanxi 710072, China; School of Computer Science, Northwestern Polytechnical University, 127 West Youyi Road, Beilin District, Xi’an, Shaanxi 710072, China; School of Computer Science, Northwestern Polytechnical University, 127 West Youyi Road, Beilin District, Xi’an, Shaanxi 710072, China; School of Life Science and Technology, Northwestern Polytechnical University, 127 West Youyi Road, Beilin District, Xi’an, Shaanxi 710072, China; School of Life Science and Technology, Northwestern Polytechnical University, 127 West Youyi Road, Beilin District, Xi’an, Shaanxi 710072, China; School of Computer Science, Northwestern Polytechnical University, 127 West Youyi Road, Beilin District, Xi’an, Shaanxi 710072, China; Department of Orthopedic Surgery, Orthopedics Oncology Institute of Chinese PLA, Tangdu Hospital, Air Force Military Medical University, 1 Xinsi Road, Baqiao District, Xi’an 710038, China; School of Life Science and Technology, Northwestern Polytechnical University, 127 West Youyi Road, Beilin District, Xi’an, Shaanxi 710072, China

**Keywords:** dual-stage, reinforcement learning, large language model, molecule optimization

## Abstract

As a critical step in drug discovery, molecule optimization aims to improve specific properties of lead compounds. Inspired by isosteres (i.e. molecular substructures with similar reactive electron shells), existing AI-based methods have been proposed for molecule optimization. However, they often rely on available matched molecular pairs (MMPs), thus facing two essential challenges. First, the limited diversity of available MMPs leads to biased training and a lack of novelty in the optimized molecules, in both single-objective (SO) and multi-objective (MO) optimization. Second, the low number of available MMPs leads to limited generalization in MO optimization. To overcome the limitations of MMPs, we propose DFRL-Mol, a novel dual-stage reinforcement learning framework. It consists of three main modules: the Structure–Property Analyst (SPA), the Decorator (DCR), and Curriculum-guided Dual-Stage Policy Optimization (CDSPO). SPA and DCR are two LLM-based models, which are responsible for locating the substructures to be optimized and inferring their appropriate isosteric replacement, respectively. CDSPO leverages curriculum-guided reinforcement learning to facilitate collaboration between the two models. DFRL-Mol ensures the generation of diverse molecular representations while effectively overcoming the issues of bias and data sparsity associated with MMPs. The comparisons with state-of-the-art methods demonstrate the enhanced performance of DFRL-Mol in multiple scenarios of molecule optimization, including SO optimization, substructure-constrained (SC) SO optimization, MO optimization, and MO molecule generation. More importantly, detailed analysis of experimental results demonstrates that the models’ behavior is consistent with chemical intuition.

## Introduction

Lead optimization in drug discovery is a critical task, where isosteric replacement is one of the widely employed strategies [1]. Isosteres are defined as functional groups or molecular fragments that possess similar physicochemical properties and biological activities [1]. Isosteric replacement can achieve simultaneous optimization of activity and druggability with only minor structural perturbations. In recent years, with the rapid development of deep learning, using matched molecular pairs (MMPs) to achieve isosteric replacement has gradually become one of the mainstream paradigms in the field of molecule optimization.

Early works focused on an implicit “one-step generation” paradigm. They typically treat molecule optimization as a translation task [2] and perform supervised learning based on MMPs. In other words, a molecule with suboptimal properties (i.e. the source language) is “translated” into a molecule with improved properties (i.e. the target language). For example, some studies represent molecules as SMILES strings and employ a Seq2Seq architecture for end-to-end transformation [3]. Subsequent works focus on the graph structures of molecules, predicting the modified molecule via graph-to-graph translation models [4]. However, these methods, relying on available MMPs entries, face two core challenges that limit their performance and scope of application. The first challenge concerns the limited coverage of chemical space, which is often restricted to variations around known privileged scaffolds [5], and the inherent bias in MMPs data (e.g. typically cleaving molecules only at acyclic single bonds [6]). The second challenge is data sparsity in MO property optimization. A common alternative solution is to combine multiple SO datasets for training, but it often leads to conflicting strategies between different optimization goals.

Concurrently, the unconstrained exploration paradigm based on generative models has gradually emerged [7–10]. Representative techniques are usually based on Variational Autoencoders (VAEs) [11], Generative Adversarial Networks (GANs) [12, 13], or Flow-based Models [14]. The core idea is to learn the latent distribution of the chemical space from large-scale unlabeled molecular datasets, allowing them to implicitly learn chemical rules. However, existing methods still face challenges in generalizable, stable, interpretable, and multi-objective lead optimization. To achieve the property-directed molecule optimization, reinforcement learning (RL) has been introduced [15, 16]. Within the RL framework, the generative model acts as the “agent” to explore the chemical space, while one or more property predictors constitute the “environment” and provide reward signals. Although this paradigm has shown potential in exploring novel chemical structures, it still faces new challenges. To ensure the chemical validity of the generated molecules, limited human-defined rules are often incorporated [9]. This requirement causes these models to fail in resolving data bias.

In recent years, a knowledge-transfer paradigm based on large language models (LLMs) has emerged [17, 18]. Since LLMs pre-trained on massive volumes of SMILES texts can implicitly learn chemical syntax rules and structural knowledge, they offer the potential to overcome the biases of previous rule-based or MMPs-dependent models [19–21]. However, when applied to molecule optimization, this paradigm encounters the core challenge of how to perform target property-oriented optimization of molecule structures. Most studies still adopt the supervised fine-tuning (SFT) approach, which inevitably relies on paired data (e.g. MMPs). This reliance means they inherit the fundamental limitations of the first paradigm, failing to exploit the advantages of their pre-training. Although a few studies attempting to integrate reinforcement learning have indicated a promising direction [17], it remains an open question about how to leverage the prior knowledge of LLMs while enabling efficient, stable, and multi-objective exploration.

To address the aforementioned issues, this paper proposes a novel dual-stage reinforcement learning framework, which accounts for multiple scenarios of molecule optimizations. It decouples the complex optimization task into two sub-tasks: “localization” and “replacement.” This approach allows the models to learn diverse molecular representations from extensive unsupervised datasets, thereby avoiding the intrinsic biases and overcoming the data sparsity limitations associated with MMPs. In addition, it designs a Curriculum-guided Dual-Stage Policy Optimization (CDSPO) module, to learn the relationship between isosteric substructures and molecular properties within molecular structures by coordinating the collaboration between the above two models through reinforcement learning.

## Methods

### Problem definition

Molecule optimization is the process of iteratively modifying an initial molecular structure to enhance its specific properties under a given set of constraints. This process is formulated as a sequential decision problem and formalized as a Markov Decision Process (MDP). Let $\mathcal{M}=\left\{\dots, {m}_t,\dots \right\}$ be the state space, $\mathcal{A}=\left\{\dots, {a}_t,\dots \right\}$ be the action space, $\mathcal{P}$ be the state transition function, and $\mathcal{R}$ be the reward function. An MDP can be defined as


(1) \begin{equation*} MDP=\left\langle \mathcal{M},\mathcal{A},\mathcal{P},\mathcal{R}\right\rangle . \end{equation*}

In the context of molecule optimization, $\mathcal{M}$ is the set of all valid molecular structures (states), and $\mathcal{A}$ represents the set of chemical structural modification operations that can be performed on the molecule of interest. More specifically, this work decouples the optimization task into two sub-tasks: “localization” and “replacement,” which are implemented by two models: a Structure–Property Analyst (SPA) and a Decorator (DCR), respectively. Thus, $\mathcal{A}$ can be defined as a composite action space as follows


(2) \begin{equation*} \mathcal{A}={\mathcal{A}}_{SPA}\times{\mathcal{A}}_{DCR}, \end{equation*}

where ${\mathcal{A}}_{SPA}$ is the site-positioning action selected by the localization model identifying the fragment or functional group for replacement, and ${\mathcal{A}}_{\mathrm{DCR}}$ is the fragment replacement action selected by replacement model generating an isostere to substitute the site selected by ${\mathcal{A}}_{\mathrm{SPA}}$. The state transition function $P\left({m}_{t+1}|{m}_t,{a}_t\right)$ describes the probability of transitioning from the current molecular structure ${m}_t$ to a novel molecular structure ${m}_{t+1}$ after executing action ${a}_t$ (i.e. localizing or replacing substructure). The reward function $R\left({m}_t,{a}_t,{m}_{t+1}\right)$ evaluates the executed action turning ${m}_t$ to ${m}_{t+1}$.

We employ reinforcement learning to optimize the policy ${\pi}_{\theta }$ (${a}_t\sim{\pi}_{\theta}\left(\bullet |{m}_t\right)$). Unlike supervised learning, which typically minimizes cross-entropy loss, our objective is to maximize the expected cumulative reward, formally defined as:


(3) \begin{equation*} J\left(\theta \right)={\mathbb{E}}_{\pi_{\theta }}\left[\sum_{t=0}^T\mathcal{R}\left({m}_t,{a}_t,{m}_{t+1}\right)\right], \end{equation*}

by maximizing this objective, the agents align their strategies with reward signals, guiding the evolution of molecular structures toward high-value regions.

To solve the above MDP, this work proposes a novel **D**ual-Stage **R**einforcement **L**earning framework for **Mol**ecule optimizations (DFRL-Mol) in the following sections.

### Overview of DFRL-Mol

DFRL-Mol is a collaborative dual-stage architecture consisting of three core modules: the SPA, the DCR, and the CDSPO. The framework decouples the complex molecule optimization task into two sequential sub-tasks: “localization” and “replacement,” which are executed by the SPA and DCR, respectively.

The SPA acts as the “Architect,” simulating the structural insight of a medicinal chemist to identify optimization sites. Its input is a lead molecular SMILES string combined with an optimization objective, and its output is a masked SMILES fragment indicating the localized region for modification. The DCR acts as the “Builder,” responsible for inferring appropriate isosteric replacements for the masked region. It takes the masked fragment generated by the SPA as input and reconstructs it into a complete, chemically valid molecule. Its objective is to execute structural modifications that enhance target properties.

While the LLMs provide the generative capabilities, CDSPO coordinates their collaboration. By utilizing a curriculum-guided reinforcement learning strategy, CDSPO enables the LLMs to learn the complex relationship between isosteric substructures and molecular properties. It dynamically updates the policy to ensure that the diverse molecular representations generated by the LLMs evolve towards high-value regions of the chemical space.

### Preliminary training of models

**Figure 1:**
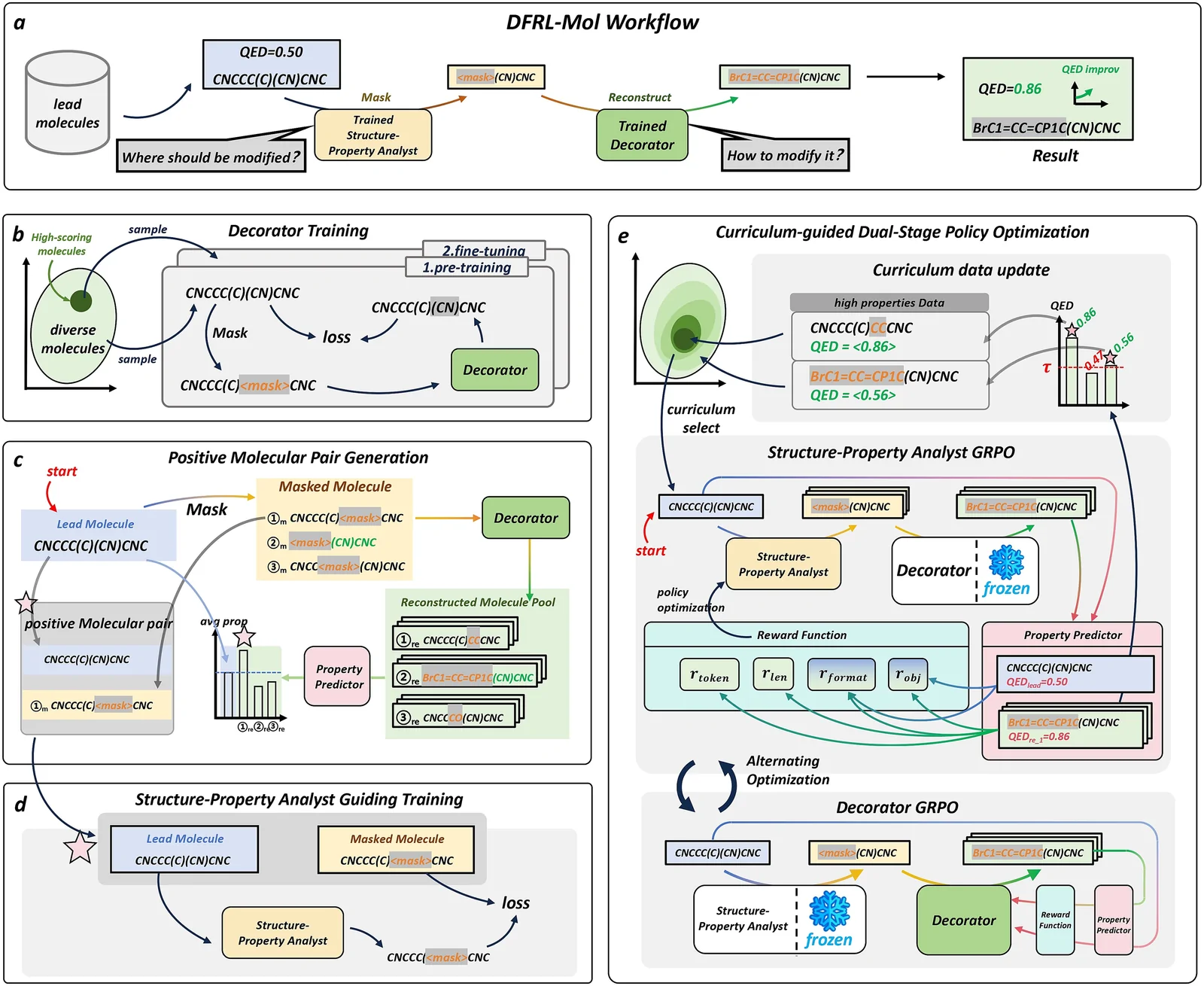
Schematic illustration of the DFRL-Mol framework. (a) Optimization stage. One molecule generates multiple candidate results. Ultimately, an optimal result is obtained. (b) DCR training. DCR performs MLM on unlabeled datasets. Pre-training and fine-tuning are conducted on diverse molecular datasets and datasets with high property scores, respectively. (c) Molecular pair generation. Based on the DCR reconstruction results, we identified sites that contribute to process optimization. Positive samples from this process are collected as molecular pair data, denoted as (lead molecule, masked molecule). (d) Using the data generated in the previous step, the SPA is initially guided. (e) CDSPO stage, where an iterative data augmentation strategy based on curriculum learning is introduced. It modulates the training difficulty by adjusting the ratio of molecules across increasing property gradients to guide the model’s continuous exploration within high-value chemical space. During training, the two models are frozen alternately to ensure training stability. Finally, QED in figure is only used for demonstration.

The preliminary training is designed to acquire diverse molecular representations and achieve preliminary objective guidance. The overall workflow comprises three stages: DCR Training (Fig. 1b), Positive Molecular Pair Generation (Fig. 1c), and SPA Guiding Training (Fig. 1d). In our experiments, Qwen2.5-0.5B-Instruct serves as the backbone for both models. The detailed hyperparameters and training configurations are provided in Supplementary Material S1.

#### DCR training

The DCR adopts a self-supervised masked reconstruction strategy to capture fundamental chemical rules and structural information. First, the model is pre-trained on large-scale unsupervised molecular data to establish a diverse structural prior. Following this, the model undergoes fine-tuning using a dataset of high-scoring molecules, these molecules are screened from the pre-training dataset using a property predictor. This phase drives the convergence of the model’s output distribution toward the high-value regions of chemical space. Throughout this entire training process, masking and reconstruction are core steps. Their purpose is to create controlled, local information loss. To this end, we perform span masking on the SMILES strings at the character level. Our strategy is to first randomly select a starting position and then determine a contiguous character span. The length of this span is restricted to no more than 30% of the total SMILES string length to avoid excessively large modifications. Subsequently, all characters within this span are replaced by a single [mask] token.

This approach prevents the model from inferring the length of the original fragment from the number of [mask] tokens. Formally, let ${\theta}_D$ denote the parameters of the DCR. Given a dataset of unlabeled molecules, the training objective is to minimize the negative log-likelihood of reconstructing the original fragment *f* given the masked context $\overset{\sim }{m}$:


(4) \begin{equation*} {\mathcal{L}}_{DCR}\left({\theta}_D\right)=-{\mathbb{E}}_{\left({m}_{mask},f\right)\sim{D}_{unlabeled}}\left[{log}{P}_{\theta_D}\left(f|{m}_{mask}\right)\right], \end{equation*}

where ${\theta}_D$ denotes the trainable parameters of the DCR, and ${D}_{unlabeled}$ represents the large-scale dataset of unlabeled molecular structures. *m* refers to a complete molecular sample drawn from the dataset. Through this process, the DCR learns a conditional probability distribution that implicitly encodes the intrinsic chemical language, enabling it to generate chemically valid fragments ${f}_{new}$ coherent with missing fragments.

#### SPA guiding training

In the SPA guiding training, we aim to identify sites that contribute to property optimization. To achieve this without manual annotation, we utilize the generative capability of the pre-trained DCR to construct a dataset of “positive molecular pairs” (Fig. 1c). This generated dataset is then employed to explicitly guide the SPA via supervised training (Fig. 1c).

Specifically, for each lead molecule, we generate multiple masked variants by randomly masking various structural fragments. These masked variants are then input into the DCR for reconstruction. This process is formalized as follows:


(5) \begin{align*} {D}_{gen}=\left\{\left(m,{\left\{{f}_i^{mask}\right\}}_i^K,{\left\{m{\prime}_i\right\}}_i^K\right)\right\}, \end{align*}

where ${D}_{gen}$ denotes the generated dataset, ${\left\{{f}_i^{mask}\right\}}_i^K$ represent *K* masked variants, ${\left\{m{\prime}_i\right\}}_i^K$ represent molecules reconstructed by the DCR. A property predictor then evaluates the reconstructed molecules. We filter these samples to retain only the successful modifications—instances where the reconstructed molecule exhibits improved properties compared to the original lead molecule, namely:


(6) \begin{equation*} {D}_{pos}={\left\{\left({m}_i,{f}_i^{mask}\right)|S\left(m{\prime}_i\right)>S(m)\right\}}_i^K, \end{equation*}

where ${D}_{pos}$ is the set of positive molecules, and $S\left(\bullet \right)$ serves as the property evaluation function. These successful cases are collected as positive pairs $\left({m}_i,{f}_i^{mask}\right)$, where *m* represents the original molecule and ${f}_i^{mask}$ represents the masked original molecule. This process effectively converts the DCR’s implicit structural knowledge into explicit supervision signals for the SPA.

Finally, we conduct guided training for SPA using the data generated in the previous step. The goal is to equip the SPA with the initial capability to identify high-potential sites before reinforcement learning begins. This process allows the SPA to explicitly learn from the high-likelihood regions (the “successful” modifications) within the distribution, acting as a bridge between self-supervised learning and the subsequent reinforcement learning stage.

The SPA is treated as a conditional policy model that selects a structural fragment to be optimized. We fine-tune the SPA via supervised learning to maximize the likelihood of selecting the “successful” sites identified in the pair generation stage. The optimization objective is defined as:


(7) \begin{equation*} {\mathcal{L}}_{SPA}\left({\theta}_S\right)=-{\mathbb{E}}_{\left(m,{m}_{mask}\right)\sim{D}_{pos}}\left[{log}{P}_{\theta_S}\Big({m}_{mask}|m,T\right], \end{equation*}

where ${\theta}_S$ represents the trainable parameters of the SPA. ${D}_{pos}$ denotes the positive dataset constructed in the previous stage, containing pairs of molecules and their corresponding optimal optimization sites. *T* indicates the target property provided as a condition token. Minimizing this loss ensures that the SPA learns an effective site-positioning policy that aligns with the global optimization goals, thereby accelerating the convergence of the subsequent training.

### Curriculum-guided dual-stage policy optimization

After the pre-training phase, LLMs provides an initial ability to explore chemical space. To enable the LLMs to learn how to obtain desired molecular properties (i.e. an MDP problem described in Section 2.1), we design CDSPO under the framework of reinforcement learning [22]. The initial policies for CDSPO are directly inherited from the models optimized in the preliminary stages, equipping the agents with initial optimization and exploration capabilities.

To optimize the policy efficiently, we employ Group Relative Policy Optimization (GRPO) [23], a reinforcement learning algorithm specifically tailored for Large Language Models. Unlike the standard Proximal Policy Optimization (PPO) which relies on a value function (Critic) to estimate advantages, GRPO eliminates the need for an additional Critic network. Instead, it estimates the baseline from a group of outputs generated from the same input, thereby reducing computational overhead and training instability.

Specifically, for each molecular state *m*, the policy generates a group of action outputs $\left\{{\mathrm{a}}_1,{\mathrm{a}}_2,\dots, {\mathrm{a}}_{\mathrm{G}}\right\}$. The optimization objective is to maximize the surrogate objective function:


(8) \begin{equation*} {\mathcal{L}}_{GRPO}=\mathbb{E}\left[\frac{1}{G}\sum_{i=1}^G{\min}\left(r\left(\theta \right){A}_i, clip\left(r\left(\theta \right),1-\epsilon, 1+\epsilon \right){A}_i\right)\right], \end{equation*}

where *ɛ* is the clipping hyperparameter. Let $r\left(\theta \right)$ denote the probability ratio between the current policy and the old policy:


(9) \begin{equation*} r\left(\theta \right)=\frac{\pi_{\theta}\left({a}_t|{s}_t\right)}{\pi_{\theta_{old}}\left({a}_t|{s}_t\right)}. \end{equation*}

The advantage function ${\mathrm{A}}_{\mathrm{i}}$ is computed by normalizing the rewards within the generated group, as defined in Eq. (10):


(10) \begin{equation*} {A}_i=\frac{\left({R}_i- Mean\left(\left\{{\mathcal{R}}_1,{\mathcal{R}}_2,\dots, {\mathcal{R}}_G\right\}\right)\right)}{std\left(\left\{{\mathcal{R}}_1,{\mathcal{R}}_2,\dots, {\mathcal{R}}_G\right\}\right)}. \end{equation*}

The calculation of the advantage function relies on a MO reward function, which directly determines the model’s learning direction and is composed of a weighted sum of the following components:


(11) \begin{align*} \mathcal{R}\left({m}_{lead},{m}_{output}\right)=\,& rewar{d}_{obj}\left({m}_{lead},{m}_{output}\right)+rewar{d}_{token}\left({m}_{output}\right)\nonumber\\ &+ rewar{d}_{format}\left({m}_{output}\right)+ rewar{d}_{len}\left({m}_{output}\right). \end{align*}

Here, $rewar{d}_{obj}$ is the target reward, which, as the core reward signal, directly reflects the degree of optimization of the generated molecule with respect to the target properties; $rewar{d}_{token}$ is the character constraint, which prevents the model from generating unexpected characters; $rewar{d}_{format}$ is the format constraint, which ensures the model’s output conforms to the predefined interaction template, maintaining the stability of the generation process; and $rewar{d}_{len}$ is the length constraint, which penalizes the generation of excessively long or short molecules, keeping the molecular size within a reasonable range. Specifically, aside from $rewar{d}_{obj}$, all other components function as binary indicators, yielding 1 if the requirement is met and 0 otherwise. $rewar{d}_{obj}$ is defined as follows:


(12) \begin{equation*} rewar{d}_{obj}=\left(1+S\left({m}_{output}\right)-S\left({m}_{lead}\right)\right)\cdotp{\mathbf{1}}_{\left\{S\left({m}_{lead}\right)<S\left({m}_{output}\right)\right\}}. \end{equation*}

To address the issue of reward sparsity caused by the lack of high-quality data, DFRL-Mol introduces the Curriculum Learning (CL) method [24, 25]. First, throughout the training process, the framework continuously screens all molecules generated by the LLMs, selecting elite molecules that exhibit performance on target properties to maintain a curriculum experience pool. The curriculum experience pool is updated continuously throughout training. This mechanism facilitates the gradual construction of an increasingly enriched sample library of high-performance molecules. Formally, let ${D}_{curr}$ denote the dynamic curriculum experience pool initialized with high-quality data. For a set of molecules ${M}_{gen}$ generated by the LLMs, we screen for “elite” candidates based on a threshold. The update rule for the curriculum pool is defined as:


(13) \begin{equation*} {D}_{curr}\leftarrow{D}_{curr}\cup \left\{m\in{M}_{gen}|S(m)>\tau \right\}, \end{equation*}

where $S\left(\bullet \right)$ is the property evaluation function. Second, to address the increasing difficulty of further optimizing high-scoring molecules, during training, we categorize molecular properties into distinct gradients, regarding the further optimization of high-scoring molecules as a more challenging task compared to low-property ones. By dynamically adjusting the ratio of date, we modulate the training difficulty from easy to hard. Specifically, we adjust the data composition at fixed training intervals, as detailed in Table 1. This mechanism facilitates the gradual accumulation of high-performance samples, thereby guiding the models to search and optimize within high-value regions of the chemical space. The complete optimization procedure is formally described in Fig. 1e. To ensure training stability, the SPA and DCR are alternately frozen and optimized every 500 steps, beginning with the optimization of the SPA. The complete training pipeline is summarized as a pseudocode in Supplementary Material S3.

CDSPO aims to guide the models towards alignment with optimization objective, encouraging the model to collaboratively explore effective and innovative pathways, thereby ensuring their strategies are jointly oriented with the ultimate optimization goal. For SPA, when an inappropriate site is selected (e.g. a critical pharmacophore), the DCR is more likely to fail in optimization. Consequently, the reward function will return a negative reward signal to penalize the prior site selection by SPA, thereby prompting it to learn a localization strategy that is more consistent with chemical intuition and possesses optimization potential. For DCR, the reward signal provides it with a clear optimization gradient. During the preliminary training phase, the objective of the DCR is structural reconstruction (“to be identical to the original molecule”). In contrast, during the reinforcement learning phase, its objective is reshaped to property enhancement (“to be better than the original molecule”), which guides its strategy to shift from mere structural recovery to creative structural modifications oriented towards improving the target properties.

## Results

### Tasks and evaluation

In this section, we evaluate DFRL-Mol in multiple scenarios, including two scenarios of SO optimizations (i.e. regular SO optimization and substructure-constrained SO optimization (SC-SO)), a scenario of multi-objective optimization (i.e. MO optimization), and a scenario of MO-constrained molecule generation. The SO optimization tasks include regular SO optimization task and substructure-constrained SO optimization task. These benchmarks not only encompass the standard practices in the field but also simulate the complex challenges in real-world drug discovery.

#### Properties

SO optimization includes the optimization of QED, logP, and DRD2, as well as SC optimization; MO optimization includes the joint optimization of QED & DRD2, and the generation under the condition of GSK3β & JNK3, and GSK3β & JNK3 & QED & SA, respectively. The core properties involved in these tasks are defined as follows:

- QED (Quantitative Estimate of Drug-likeness): A quantitative assessment of drug-likeness, with a value ranging from 0 to 1. A higher value indicates better drug-likeness.
- logP: The logarithm of the octanol–water partition coefficient, used to measure the lipophilicity of a molecule.
- DRD2: The activity against the Dopamine D2 receptor, a property often used to evaluate the biological activity of molecules intended as antipsychotic drugs.
- GSK3β and JNK3: Respectively, Glycogen Synthase Kinase-3β and c-Jun N-terminal Kinase-3. These are two important kinase targets commonly studied in drug research for Alzheimer’s disease.
- SA (Synthetic Accessibility) Score: A score used to estimate the ease of synthesis for a molecule. A lower value indicates that the molecule is easier to synthesize.

#### Metrics

We employ distinct metrics depending on the task type.

**For Optimization Tasks:**

- Success Rate (prop ≥ k): The proportion of optimized molecules for which the target property meets or exceeds a given threshold k.
- Improvement Rate (Δprop ≥ k): The proportion of optimized molecules for which the improvement in the target property is greater than a given threshold k.
- Average Improvement (imprv): The average improvement in the target property for the optimized molecules.

**For Generation Tasks:**

- Average Property Score (APS): The average value of the target properties for the top-K generated molecule: $APS=\frac{1}{\mid{\mathcal{G}}_K\mid}\sum_{x\in{\mathcal{G}}_K} Score(x)$, here, *Score(x)* is the target property value of a molecule *x*. For MO tasks, it is typically the result of a weighted sum of multiple properties or a scalarization function. ${\mathcal{G}}_K$ represents the subset of the top-K molecules from the set of generated molecules, ranked according to the target property function. The Synthetic Accessibility (SA) score is calculated according to the formula, where *μ* and *σ* are empirical parameters:
  \begin{align*} {SA}_{norm}={\exp}\left(-\frac{1}{2}{\left(\frac{{\max}\left( SA,\mu \right)-\mu }{\sigma}\right)}^2\right) \end{align*}
- Novelty (Nov): The proportion of molecules among the generated Top-K molecules that are not in training set: $\mathrm{Nov}=\frac{1}{\mid{\mathcal{G}}_K\mid}\sum_{x\in{\mathcal{G}}_K}\mathbb{I}\left(x\notin{\chi}_{train}\right),$where $\mathbb{I}\left(x\notin{\chi}_{train}\right)$ is an indicator function that determines if a molecule *x* is absent from the training set ${\chi}_{train}$.

### Data collection

All training stages in this study utilized a unified data source derived from a subset of the ZINC drug dataset, from which a subset of 1 million unlabeled data points was sampled as training data. All data have been made publicly available in the code repository. Therein, all molecules are represented using the SMILES notation.

**Guiding training of SPA:** a total of 50 000 successful candidates were sampled.

**Pre-training of DCR:** we utilized a randomly sampled dataset consisting of 1 000 000 molecules.

**Fine-tuning of DCR:** The phase involved a dataset of 200 000 molecules exhibiting high property scores.

**CDSPO:** the optimization targets were stratified into five difficulty levels. During the training process, high-quality generated molecules satisfying the specific criteria of a level were dynamically incorporated into the corresponding dataset to enrich the training pool. Subsequently, every 10 000 steps (one stage), we adjust the composition of the training data by increasing the proportion of hard samples, while the total dataset size remains constant at 200 000. See Table 1 for details.

**Table 1:** Distribution of samples across five levels during training.

Ratio(%)	Level-1 data	Level-2 data	Level-3 data	Level-4 data	Level-5 data
Stage-1	40	25	15	15	5
Stage-2	30	25	20	15	10
Stage-3	20	20	20	20	20
Stage-4	10	15	20	25	30
Stage-5	5	10	15	25	45

**Testing:** The testing data configuration depends on the task type. For optimization tasks, the data was sourced from the dataset provided by You et al. [15]. Specifically, for SC-SO tasks, this source set was filtered to ensure that the starting molecules possessed the substructures targeted for retention. In contrast, for generation tasks, a single molecule was adopted as the starting point. To ensure the fairness of the evaluation, Supplementary Material S10 provides a detailed overlap analysis between training and test sets, along with validation results on a non-overlapping test set after removing similar molecules.

### Experimental setups

**Molecule Optimization:** the molecule optimization benchmarks are designed to evaluate a model’s ability to enhance specific properties by modifying chemical structures while maintaining structural similarity to the original molecule. This benchmark is formulated as a constrained optimization problem, where the Tanimoto similarity coefficient (*δ*) serves as a key constrain. Specifically, during the optimization process, the similarity score between each generated molecule and the lead molecule is required to be no lower than the similarity threshold *δ*. This approach is grounded in the Similar Property Principle (SPP), which posits that structurally similar molecules tend to exhibit similar molecular properties [26]. By enforcing this similarity, the model is restricted to the local “chemical neighborhood” of the lead compound, ensuring that key structural features and the core scaffold are preserved during the optimization process. Molecules violating the Tanimoto threshold are discarded during optimization. In the SO optimization task, we established two difficulty levels: a relaxed constraint (*δ* = 0.4) to evaluate the model’s breadth of exploration, and a strict constraint (*δ* = 0.6) to assess its capacity for precise fine-tuning without disrupting the core structure. For this task, we employed a multi-step iterative optimization strategy, maintaining a molecular population of size 20 at each step for a total of 20 iterations. We compared DFRL-Mol against state-of-the-art methods, including HierG2G [4], Modof [27], FOMO [28] and Prompt-MolOpt [29]. Furthermore, to more closely emulate practical drug discovery, a SC optimization benchmark was also introduced. This benchmark evaluates the model’s ability to perform optimization while preserving key substructure.

**Molecule Generation:** In the MO molecule generation task, the primary focus is on evaluating the model’s de novo design capability—that is, its ability to generate entirely new molecules that satisfy multiple property constraints, starting from a given chemical scaffold. This task is necessary for discovering novel chemical series and exploring uncharted chemical space. We adopted an experimental protocol consistent with that of other state-of-the-art works, such as InversionGNN and DST [30]. The tasks include a dual-objective generation task (targeting activity against GSK3β and JNK3) and a four-objective generation task (additionally including QED and SA). In this task, the model begins with a starting scaffold and generates a set of candidate molecules over 50 iterations (with a population size of 100), after which the top 100 performing molecules (Top-100) are subjected to a final evaluation.

### SO molecule optimization

#### Regular SO task

To investigate the constrained SO optimization capability of DFRL-Mol, we evaluated its performance in optimizing three metrics (QED, DRD2, and logP) subject to Tanimoto similarity constraints. In addition, we compare DFRL-Mol with four representative baseline methods: HierG2G [4]: A hierarchical graph generation model. JTNN [2]: A junction tree-based variational autoencoder. Modof [27]: A modification model based on fragment removal and addition. FOMO [28]: A graph editing model that incorporates atomic importance information.

In the QED SO optimization task, DFRL-Mol demonstrated high performance, especially under stricter constraints (results are shown in Table 2). When the similarity constraint was tightened to *δ* = 0.6, the performance of all baseline models experienced a significant reduction (Modof: QED ≥ 0.9 from 66.25% to 18.62%). Under this stringent constraint, although DFRL-Mol also saw a decline, its rate of decline was relatively stable. This result demonstrates the stability of DFRL-Mol under strict structural constraints. In the logP or DRD2 SO optimization task, DFRL-Mol achieved competitive results in both tasks. We also observed that DFRL-Mol demonstrated stability under strict constraints. The above results collectively reveal an important characteristic of DFRL-Mol: DFRL-Mol does not rely on large-scale structural modifications but instead enables precise optimization within a constrained operational space, thereby achieving superior performance under strict constraints. Supplementary Materials S2 and S7 present the convergence analysis and representative case studies, respectively.

**Table 2:** Comparison of SO molecule optimization.

	Metrics	HierG2G	Modof	FOMO	DFRL-Mol
${\delta} =$ 0.4	QED ≥ 0.9↑	37.12%	66.25%	82.00%	**94.78%**
	∆QED ≥ 0.1↑	65.88%	87.62%	95.55%	**98.72%**
	logP imprv↑	3.98	5.89	6.03	**6.97**
	DRD2 ≥ 0.5↑	51.80%	88.60%	**90.20%**	86.50%
	∆DRD2 ≥ 0.2↑	70.20%	95.90%	**96.40%**	95.90%
${\delta} =$ 0.6	QED ≥ 0.9↑	17.38%	18.62%	22.95%	**54.05%**
	∆QED ≥ 0.1↑	37.25%	39.50%	52.05%	**84.75%**
	logP imprv↑	2.49	3.14	3.37	**3.50**
	DRD2 ≥ 0.5↑	10.50%	28.90%	28.08%	**40.70%**
	∆DRD2 ≥ 0.2↑	19.70%	45.30%	48.16%	**55.00%**

#### Substructure-constrained SO task

The SC molecule optimization experiments are designed to assess the model’s ability to optimize target molecular properties while preserving key structural features. This study introduces two types of constraints: scaffold preservation (represented by the Benzene ring) and pharmacophore retention (represented by the Hydroxyl group). Consequently, the Model is required to maintain the presence of pharmacophore in the molecule optimization. Considering stricter constraints, we increased the number of iterative optimization rounds to 50 for this task. Finally, the Tanimoto similarity constraint maintained to ensure the generated molecules stay within the chemical neighborhood of the lead compound.

We compared the performance of DFRL-Mol under constrained conditions against its unconstrained baseline. These results demonstrate DFRL-Mol’s capability to perform optimization under the dual constraints of similarity and substructures. As shown in Table 3, although the presence of constraints restricts the optimization space, the final absolute logP values achieved by the model show a minimal gap when compared to the unconstrained baseline. In addition, among the two groups, the benzene ring is relatively favorable for increasing logP. Conversely, the hydroxyl groups are hydrophilic moieties, and preserving it inherently opposes the objective of increasing logP. Despite these conflicting chemical properties, DFRL-Mol still achieves significant property improvements. This result confirms that the substructure constraints do not fundamentally compromise the core capabilities of the model, demonstrating its robustness even when facing chemically challenging constraints.

**Table 3:** Substructure-constrained logP optimization by DFRL-Mol.

		logP imprv↑(with SC)	logP imprv↑(w/o SC)
Benzene	*δ* = 0.4	6.57	6.67
	*δ* = 0.6	3.84	3.88
Hydroxyl	*δ* = 0.4	9.14	9.31
	*δ* = 0.6	6.63	6.76

With SC and w/o SC denote optimization with and without substructure constraints (e.g. Benzene or Hydroxyl), respectively.

To validate the generalizability of DFRL-Mol across distinct optimization landscapes, we extended the evaluation to QED and DRD2. The results, presented in Tables 4 and 5, demonstrate a robust performance consistent with the logP experiments: even when the exploration space is restricted by substructure constraints, DFRL-Mol effectively enhances these complex properties. In summary, these results confirm the broad potential of DFRL-Mol in real-world molecule optimization scenarios.

**Table 4:** Substructure-constrained QED optimization by DFRL-Mol.

		QED ≥ 0.9(with SC)	*Δ* QED ≥ 0.1(with SC)	QED ≥ 0.9(w/o SC)	*Δ* QED ≥ 0.1(w/o SC)
Benzene	*δ* = 0.4	73.96%	88.96%	99.41%	99.59%
	*δ* = 0.6	41.09%	82.04%	63.44%	92.84%
Hydroxyl	*δ* = 0.4	63.30%	85.68%	99.11%	99.39%
	*δ* = 0.6	29.65%	75.87%	64.25%	92.01%

**Table 5:** Substructure-constrained DRD2 optimization by DFRL-Mol.

		DRD2 ≥ 0.5(with SC)	*Δ* DRD2 ≥ 0.2(with SC)	DRD2 ≥ 0.5(w/o SC)	*Δ* DRD2 ≥ 0.2(w/o SC)
Benzene	*δ* = 0.4	95.44%	99.02%	95.10%	99.36%
	*δ* = 0.6	58.10%	76.31%	61.64%	84.74%
Hydroxyl	*δ* = 0.4	96.87%	98.99%	98.96%	99.06%
	*δ* = 0.6	55.20%	82.29%	58.33%	81.25%

### MO molecule optimization

MO optimization aims to evaluate a model’s balancing capability when simultaneously optimizing multiple, often competing, property objectives. This experiment focuses on a MO optimization task involving DRD2 and QED. This task requires the model to enhance activity while maintaining good druggability, a trade-off between multiple properties that represents a common challenge in drug design.

**Figure 2:**
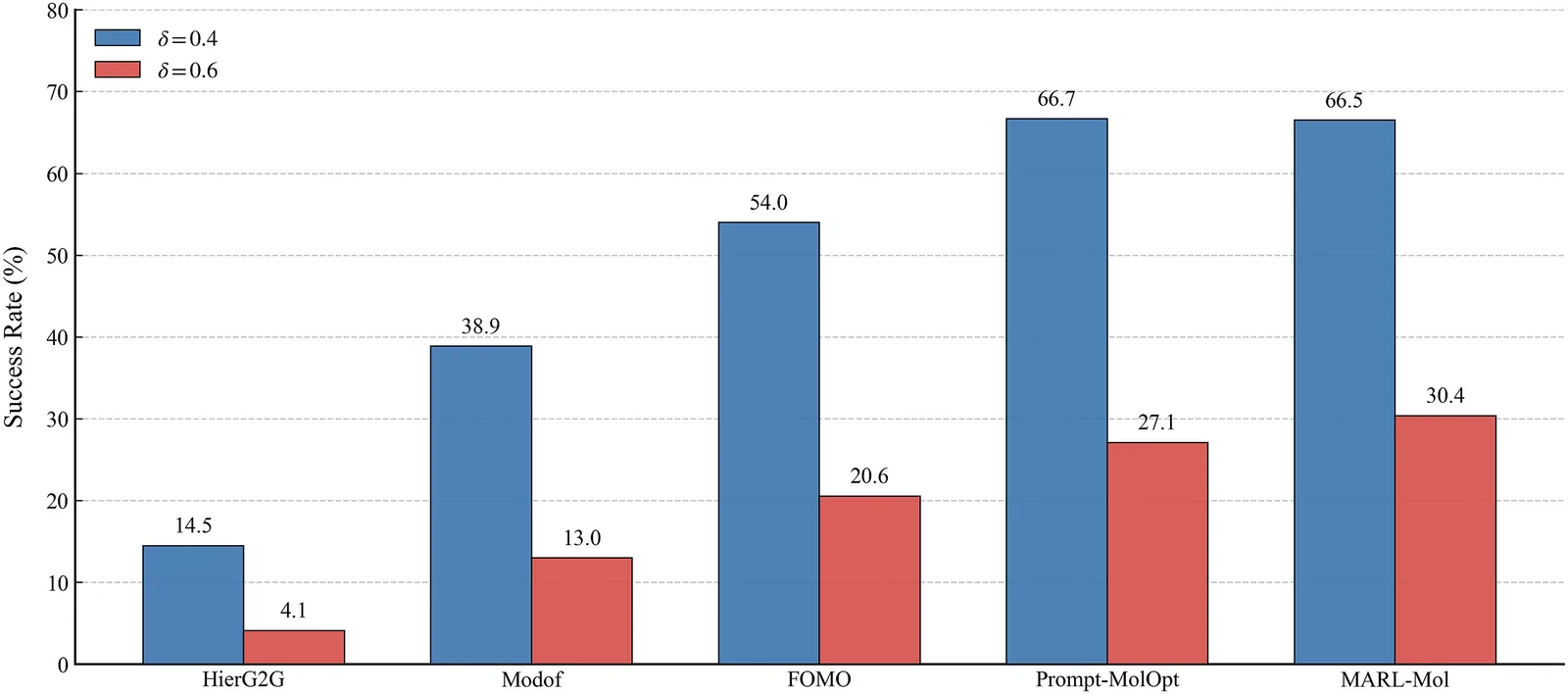
Comparison in the case of QED&DRD2 optimization. Performance comparison against state-of-the-art baseline methods, including HierG2G, Modof, FOMO, and Prompt-MolOpt [29]. The success rate is defined as the proportion of molecules in the test set that satisfy both QED > 0.6 and DRD2 > 0.5 after optimization.

As shown in Fig. 2, we evaluated the performance of DFRL-Mol under different structural similarity constraints, with the core metric being the proportion of molecules that simultaneously satisfy DRD2 ≥ 0.5 and QED ≥ 0.6, referred to as the MO success rate. These results indicate that DFRL-Mol can effectively balance multiple objectives. Detailed comparisons reveal that while DFRL-Mol exhibits a slightly lower success rate than Prompt-MolOpt under loose constraints, it surpasses Prompt-MolOpt under stricter constraints. Experimental results demonstrate DFRL-Mol possesses the capability to efficiently and optimize multiple competing objectives under strict constraints.

DFRL-Mol has achieved competitive results across all tested molecule optimization tasks. The experimental results demonstrate that the framework not only excels in single molecular property optimization but also exhibits superior success rates and robustness compared to existing methods, especially under strict, multiple constraints. A detailed analysis of optimization, including success rate convergence, property distribution evolution, and scaffold diversity throughout the optimization process, is provided in Supplementary Material S5.

### MO molecule generation

To evaluate the model’s de novo design capabilities, this experiment evaluates the ability of DFRL-Mol to generate candidate molecules that possess high biological activity, good drug-likeness, and synthetic accessibility. It aims to simulate the critical scenario of early-stage screening for novel compounds in drug discovery. GSK3β and JNK3 are critical therapeutic targets in the pathology of Alzheimer’s disease, making their simultaneous inhibition a promising strategy for neuroprotection. However, this task presents a challenge in polypharmacology: it requires the model to navigate the intersection of two distinct chemical spaces, necessitating a balance to satisfy the structural constraints of both binding pockets simultaneously. In this task, we compare our method with the following approaches: RationaleRL [31]: An interpretable generative model based on the assembly of key substructures. DST [32]: An autoregressive subgraph generation model. InversionGNN [30]: A zero-shot generation model based on latent space optimization. The experimental results, as shown in Table 6, indicate that DFRL-Mol achieved competitive performance on both tasks. Furthermore, the model achieved 100% novelty (Nov) on both tasks.

**Table 6:** Comparison of MO molecule generation.

Properties	Metric	RationaleRL	DST	InversionGNN	DFRL-Mol
GSK3β & JNK3	Nov↑	**100%**	**100%**	**100%**	**100%**
	APS↑	0.795	0.75	**0.841**	**0.841**
GSK3β & JNK3 & QED & SA	Nov↑	99%	**100%**	**100%**	**100%**
	APS↑	0.675	0.752	0.773	**0.804**

The experimental results confirm the advantage of DFRL-Mol in molecule generation tasks. The model’s higher performance in APS demonstrates that its dual-stage architecture can effectively and synergistically optimize multiple competing property objectives, showcasing strong MO coordination capabilities. Supplementary Material S9 further extends the evaluation to include ADMET-related properties, demonstrating the framework’s applicability in more drug-relevant scenarios. In summary, DFRL-Mol is not only an efficient molecule optimizer but also an effective molecule generator. This framework can effectively handle the complex trade-offs and potential conflicts among different properties. To further validate whether the optimized molecules exhibit favorable binding in 3D space, we performed molecular docking for representative candidates. The comparison between predicted scores and docking binding affinities is provided in Supplementary Material S6.

## Visualization of experimental results

### Diversity of backbones

To investigate the chemical space coverage, we performed a Bemis-Murcko scaffold analysis [33] comparing the dataset used in DFRL-Mol against a standard MMPs-derived dataset. This method abstracts molecules into their fundamental frameworks by retaining ring systems and linkers while removing side chains, serving as a robust metric to quantify structural diversity. In addition, the DFRL-Mol dataset was randomly sampled from the pre-training data, with the sample size aligned with the total volume of the MMPs dataset to facilitate a fair comparison.

As shown in Table 7, the structural compositions of the two datasets diverge substantially. Unlike the MMPs baseline, which tends to cluster around a limited set of core structures, DFRL-Mol’s dataset demonstrates a significantly flatter distribution, requiring a much larger variety of scaffolds to achieve equivalent coverage. Furthermore, our data exhibits a markedly higher scaffold-to-molecule ratio and singleton rate. These metrics collectively indicate that our dataset avoids excessive repetition of specific chemical series, reflecting superior structural diversity compared to the baseline. This suggests that the proposed framework is exposed to a broader chemical space during training, thereby reducing the risk of overfitting to “privileged scaffolds” and enabling the learning of generalized chemical transformation rules rather than memorizing modification patterns within specific, repetitive chemical series.

**Table 7:** Bemis-Murcko scaffold analysis.

Metric	MMPs train dataset	DFRL-Mol train dataset
Total molecules	287 429	**280 000**
Unique scaffolds	66 253	**136 384**
Singleton scaffolds^a^	57.4%	**84.7%**
Scaffolds for 50% coverage^b^	2968	**9038**

^a^Scaffolds that appear only once in the entire dataset, indicating high novelty.

^b^ The number of top-ranked scaffolds required to cover 50% of the dataset. A higher number indicates a flatter distribution.

### Category preference of generated bioisosteres

**Figure 3:**
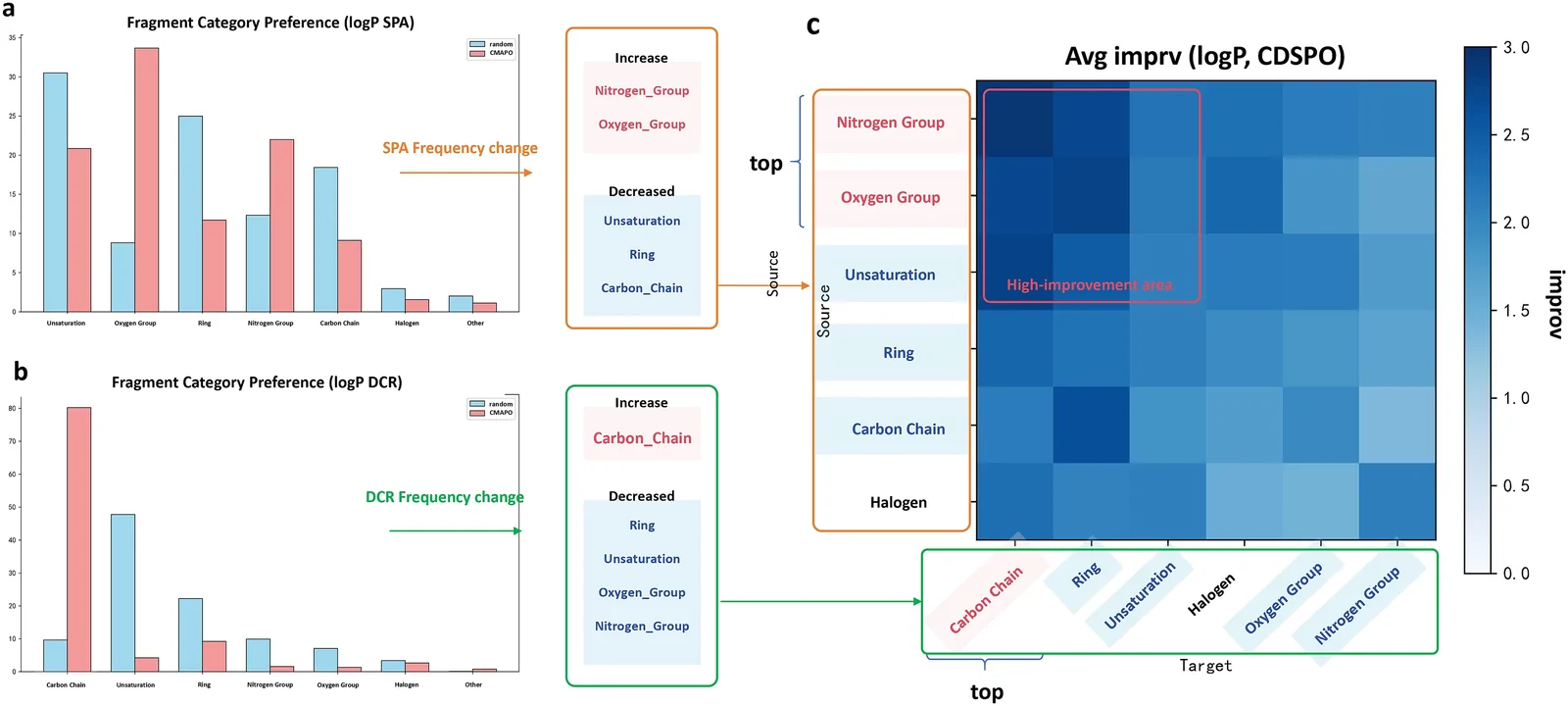
Category preference of generated bioisosteres. (a and b) Changes in the selection frequency of different chemical categories for the SPA (localization) and DCR (replacement) before and after training. Boxes indicate the categories whose selection frequency increased or decreased, respectively. (c) Performance heatmaps, different category sorted by and average improvement rate. The upper left corner represents the high-improvement area. A comparative analysis reveals that the shift in model preferences aligns with the ranking of average improvement rates.

To systematically evaluate the chemical selection preferences learned by the models, we analyzed the specific optimization trajectories of the SPA and DCR during the logP optimization process. Specifically, we examined the molecular fragments masked by SPA and those generated by DCR to investigate the model’s macroscopic structural preferences. We classified these designed bioisosteres into six major categories, prioritized as follows: Halogen > Unsaturation > Oxygen Group > Nitrogen Group > Carbon Chain > Ring. These categories cover over 97% of the molecular fragments used by the model. To capture the structural transformation dynamics, we denote the category of the fragment masked by SPA as A and the category of the fragment generated by DCR as B, simplifying the one-step optimization as “A → B.” Next, we calculated the selection frequency of these six fragment categories by the SPA and DCR during the optimization process and compared it against a baseline trained with random strategies, the experimental results are shown in Fig. 3. The results demonstrate that the models autonomously learned chemically consistent strategies to optimize logP, exhibiting a clear shift in structural preferences.

For the SPA, CDSPO training induced a strategy aligned with chemical intuition. Selection rates increased for polar categories: “Nitrogen Group” (12%–24%) and “Oxygen Group” (8%–34%), while decreasing for “Unsaturation” (39%–21%). This shift reflects a strategic prioritization: since nitrogen- and oxygen-containing functionalities (e.g. amines, carboxylic acids) typically act as primary hydrophilic centers, the model has learned to identify and target these specific sites as the “bottlenecks” for realizing logP improvement.

Regarding the DCR model, the proposed training strategy also had a distinct effect. Post-training, DCR’s policy became predominantly focused on generating fragments from the “Carbon Chain” category, this shift is chemically consistent. In the task of increasing logP, introducing aliphatic carbon chains is one of the established strategies. Although such continuous chain extending might be suboptimal for realistic drug discovery, in the context of SO optimization, this phenomenon highlights the model’s capacity to autonomously discover and exploit chemically consistent strategies driven by the reward.

Finally, we calculated and ranked the overall optimization performance for all “site-replacement” category combinations. The results in Fig. 3 show that the direction of SPA’s policy evolution is consistent with this performance ranking: the model increased its selection of the top-two performing categories, “Oxygen Group” and “Nitrogen Group,” while decreasing its selection of categories with relatively lower performance rankings. This result corroborates the preceding analysis of the policy shift, indicating that the model does not indiscriminately increase its selection of certain categories but has instead learned to prioritize marking site types that yield a logP gain. Supplementary Materials S4 and S8 provide ablation studies and quantitative analysis of transformation patterns, respectively.

### Substructure preference within generated bioisosteres

The analysis of the six major chemical categories indicates that the behavior of DFRL-Mol is, generally, consistent with established chemical optimization principles. However, despite the chemical consistency within a single category, molecular fragments with opposing contributions may still be classified under the same category. For example, the “Oxygen Group” category includes both polar functional groups with a negative contribution to logP (e.g. carboxylic acids, -COOH; alcohols, -OH) and groups that can be modified to have a neutral or positive contribution (e.g. ether linkages, -O-). Therefore, although category-level trends provide valuable directional signals, a fine-grained analysis at the specific functional group level is necessary to understand the chemical principles learned by the models. To this end, we conducted a more in-depth analysis of the specific substructures within each molecular fragment category. The analysis results demonstrate that the models have established a strategy of targeting hydrophilic moieties and substituting them with lipophilic groups.

**Figure 4:**
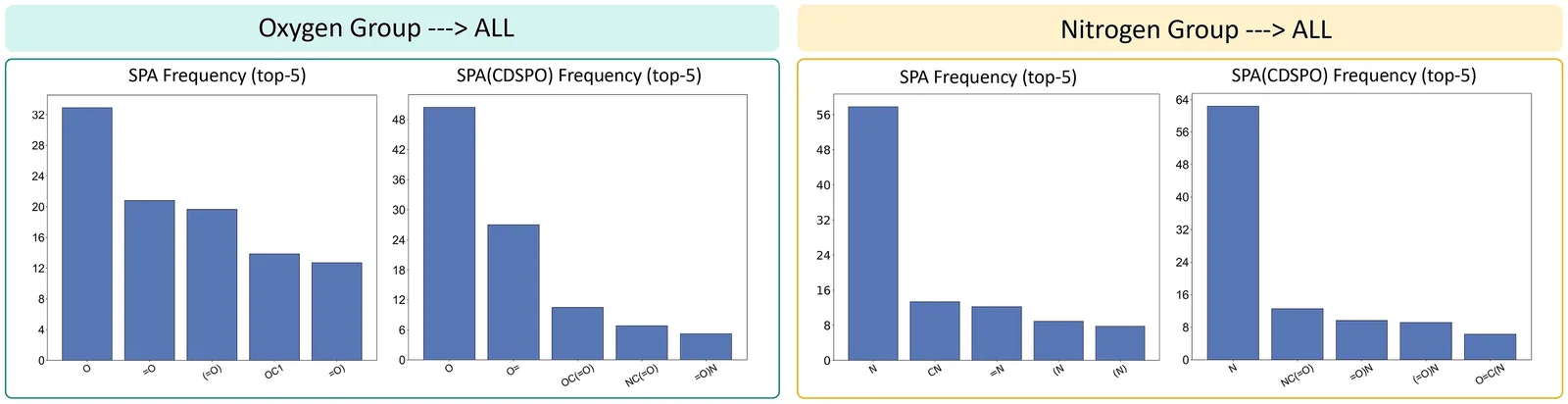
Substructure preference of the SPA within the nitrogen group and oxygen group. This figure illustrates the shift in the SPA’s selection strategy for molecular fragments within these two categories. Its focus shifts from identifying basic atoms to preferentially targeting complete polar functional groups that have a negative contribution to lipophilicity. This reflects a decision-making logic that more closely aligns with chemical reasoning.

Given that the SPA maintained high structural diversity post-training and that the “Nitrogen Group” and “Oxygen Group” exhibited the most distinct increases in selection propensity, we prioritized a fine-grained analysis of the internal composition of these two categories to investigate the drivers of this policy shift (Fig. 4). In the “Oxygen Group,” the SPA policy evolved from identifying generic units (e.g. “O,” “=O”) to specifically targeting complete polar functional groups, notably esters (OC(=O)) and carbonyl-containing hydrophilic functionalities. Similarly, within the “Nitrogen Group,” the model significantly increased its recognition of polar nitrogenous motifs. This structural specificity suggests that the model has implicitly learned to pinpoint polarity-inducing substructures, thereby facilitating the objective of logP improvement.

**Figure 5:**
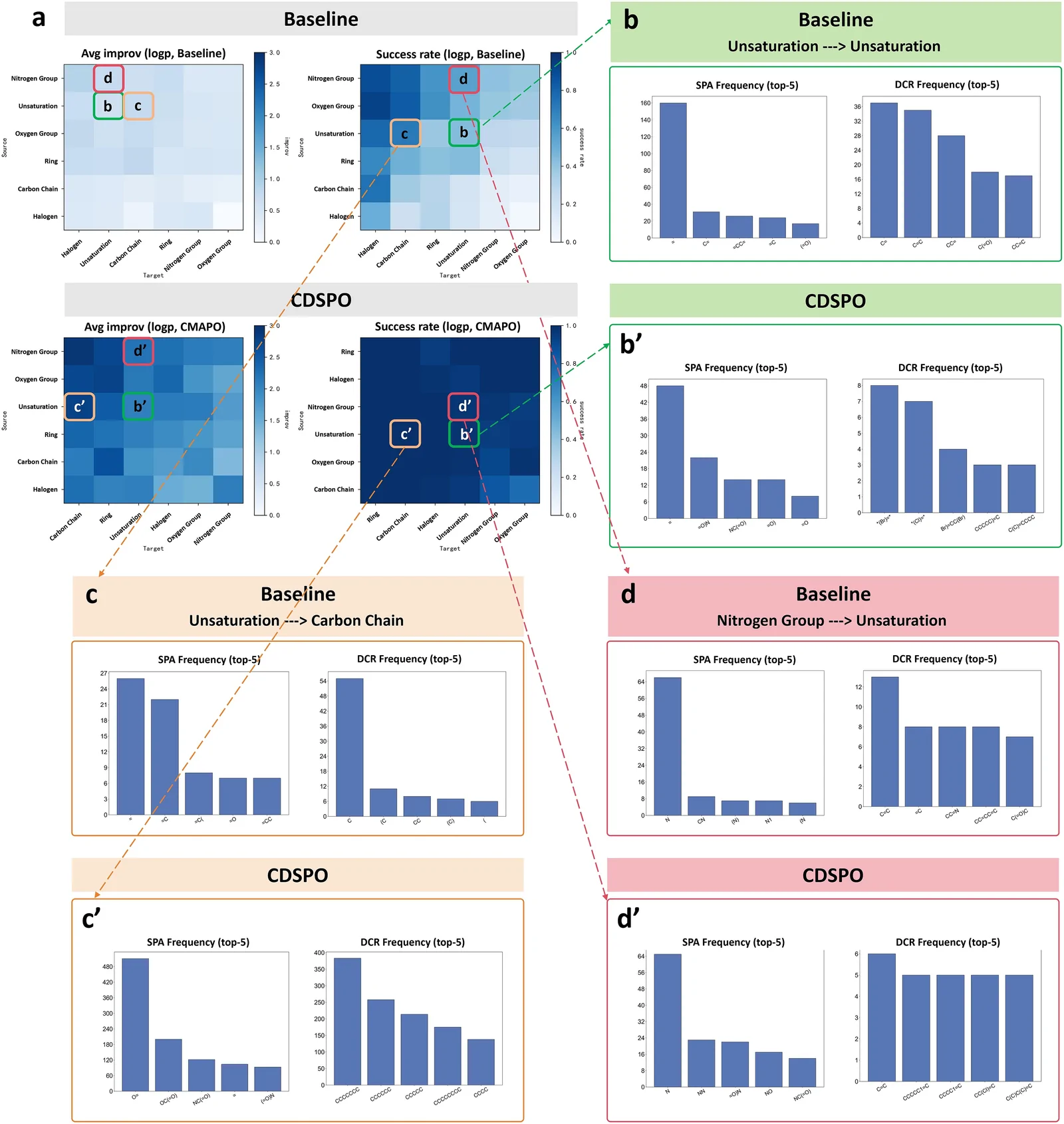
Substructure preference within generated bioisosteres. This figure details the specific changes in the internal selection preferences of the SPA (localization) and DCR (replacement) for three key replacement strategies, before and after training. (a) Shows the influence of different categories on molecular properties. In contrast, panels (b), (c), and (d) zoom in on the structural specifics by displaying the top-5 molecular fragments prioritized by SPA and DCR within specific categories. The analysis shows that SPA and DCR developed efficient and chemically intuitive synergistic strategies, the SPA learned to localize polar functional groups detrimental to the target property, while the DCR learned to replace them in chemically rational ways.

To assess the collaborative efficacy of localization and replacement, we analyzed specific category transition maps. We selected three representative fragment combinations from the aforementioned pairings, with specifics illustrated in Fig. 5.

In the “Unsaturation → Unsaturation” case, the models exhibited a consistent synergistic strategy. The SPA prioritized identifying polar amide-related sites, while the DCR shifted from generating detrimental polar groups (e.g. carbonyls) to introducing lipophilic substituents, specifically halogens and long alkyl chains. This transformation confirms the models’ ability to optimize logP through chemically rational structural modifications. Similarly, for the “Unsaturation → Carbon Chain” case, the models adopted a “hydrophilic-to-hydrophobic exchange” strategy. SPA selections concentrated on polar moieties acting as hydrophilic centers, such as aldehydes, ketones, and amides. Conversely, DCR evolved from generating generic linkers to specializing in long, straight-chain alkanes, effectively reducing polarity. Finally, in the “Nitrogen Group → Unsaturation” case, the models demonstrated a refined targeting capability. The SPA evolved from selecting generic amines to specifically targeting polar nitrogen species (e.g. nitro groups, amides) that negatively impact lipophilicity. Meanwhile, DCR corrected its pre-training inconsistency—which often generated polar amides—by shifting towards constructing extended conjugated systems and halogenated alkenes. These patterns collectively indicate that the models have successfully learned to identify and replace polar substructures with hydrophobic bioisosteres.

## Discussion

In this study, we introduced DFRL-Mol, a high-performance molecule optimization framework designed to address the critical challenges of data bias and data sparsity inherent in existing methods. By decoupling the optimization task into distinct “localization” and “replacement” phases and employing a two-stage “self-supervised pre-training & reinforcement learning” strategy, DFRL-Mol establishes a novel paradigm for molecular design. Our extensive experiments demonstrate that this approach achieves competitive performance across multiple optimization and generation benchmarks. The model exhibits robustness and generalization ability, particularly under strict structural similarity constraints and within complex multi-objective optimization scenarios. In addition to performance metrics, our analysis of the optimization trajectories reveals that models autonomously learned optimization strategies that align well with medicinal chemical intuition.

Despite the success of DFRL-Mol, we recognize several technical limitations: First, the model currently excels at small, single-step optimizations. However, tasks like de novo design or scaffold hopping require high-risk, large-scale structural exploration [34, 35]. Future work could explore more sophisticated reward mechanisms to incentivize more extensive chemical transformations. Second, the current framework is based entirely on 2D SMILES representations. This limits its application in tasks needing precise 3D spatial matching, such as structure-based drug design. A key future direction will be to integrate our dual-stage framework with emerging 3D geometric deep learning models [36]. Finally, current property predictors often cannot meet the complex demands of real-world drug development. Although molecular docking or computational methods can improve the reliability of evaluation, they have high time and computational costs, making them difficult to be directly integrated into the training process of deep learning models. Therefore, how to efficiently utilize the small but high-value feedback information from the real world will be an important exploration direction in the future.

In conclusion, the DFRL-Mol framework provides a new paradigm for AI-driven molecular design. It offers a robust solution for navigating the chemical space under strict constraints, offering new perspectives and a promising future direction for the field of molecule optimization.

**Key Points**

- A decoupled dual-stage framework is proposed for molecule optimization.
- It overcomes MMP data bias via unsupervised molecular representations.
- A CDSPO strategy facilitates efficient collaboration between LLMs.
- It achieves superior success rates while aligning with chemical intuition.

## Supplementary Material

Supplementary_material_bbag493

## Data Availability

The codes and data used in this study are available at GitHub: https://github.com/reunio/DFRL-Mol.
