# Supplementary material for: DFRL-Mol: a dual-stage framework of reinforcement learning for multi-scenario molecule optimization
Source: Brief Bioinform. 2026 Sep 10;27(5):bbag493. doi: 10.1093/bib/bbag493 (PMC13561309; doi:10.1093/bib/bbag493)
Supplement: Supplementary file 1 [file supplementary_material_bbag493.zip › Supplementary materials.docx]

DFRL-Mol: A Dual-Stage Framework of Reinforcement Learning for Multi-Scenario Molecule Optimization (Supplementary Materials)

**S1 Implementation Details**

All models were implemented using PyTorch and the Hugging Face Transformers library, trained on NVIDIA RTX A6000 GPUs. During the DCR pre-training and SPA guiding training (SFT) stages, the models were optimized using the AdamW optimizer with a learning rate of 5×10-6, a per-device batch size of 24, and a maximum sequence length of 512. The models were trained for up to 200,000 steps with a cosine learning rate scheduler. In the subsequent CDSPO reinforcement learning stage, to maintain stable policy updates and avoid catastrophic forgetting, we employed a reduced learning rate of 1×10^-6^ and a per-device batch size of 20. Specifically for the GRPO algorithm, the group size for sampling (i.e., number of generations per prompt) was set to 20, the KL penalty coefficient was set to 0.001, and the maximum generation length was restricted to 128 tokens.

**S2 Convergence Analysis**

Fig. S1 illustrates the convergence trajectories of DFRL-Mol across representative single-and multi-objective tasks. As shown, the optimization performance improves rapidly in the early stages and stabilizes at approximately 20 iterations. Continuing the process yields diminishing returns while increasing computational costs; therefore, 20 iterations were selected as a reasonable trade-off between optimization effectiveness and efficiency. Furthermore, the stable convergence under both relaxed (δ=0.4) and strict (δ=0.6) constraints confirms the model's robustness and its capability to balance competing objectives efficiently.

**Fig. S1.** Convergence analysis of DFRL-Mol. It shows the convergence line charts of four optimization tasks.

**S3 Training Pipeline of DFRL-Mol**

Algorithm 1 Training Pipeline of DFRL-Mol

Require: Unlabeled dataset $D_{unlabeled}$, Alternating frequency $N$, Curriculum stages $K$

1: Optimize DCR parameters $\theta_{D}$ on $D_{unlabeled}$ to minimize ${\mathcal{\mathcal{L}}}_{DCR}(\theta_{D})$.

2: Fine-tune $\theta_{D}$ on the high-scoring subset of $D_{unlabeled}$.

3: Generate positive molecular pairs $D_{pos}$ using DCR.

4: Optimize SPA parameters $\theta_{S}$ on $D_{pos}$ to minimize ${\mathcal{\mathcal{L}}}_{SPA}(\theta_{S})$.

5: Inheritance: Initialize policies $\pi_{\theta_{S}}\mathrm{and}\pi_{\theta_{D}}$ with the trained weights $\theta_{S}$ and $\theta_{D}$.

6: **for** stage $k=1$ to $K$ **do**

7: Update curriculum pool $D_{curr}\leftarrow D_{curr}\cup\{m\in M_{gen}\mid S(m)>\tau_{k}\}$.

8: **for** step $t=1$ to $T_{stage}$ **do**

9: Sample lead molecules $m_{t}\sim\mathcal{D}_{curr}$.

10: **if** $(t mod 2N)<N$ **then**

11: Freeze $\theta_{D}$; Unfreeze $\theta_{S}$.

12: Update $\pi_{\theta_{S}}$ to maximize ${\mathcal{\mathcal{L}}}_{GRPO}$.

13: **else**

14: Freeze $\theta_{S}$; Unfreeze $\theta_{D}$.

15: Update $\pi_{\theta_{D}}$ to maximize ${\mathcal{\mathcal{L}}}_{GRPO}$.

16: **end if**

17: **end for**

18: **end for**

**S4 Ablation Study**

The ablation study (Fig. S2) evaluated the individual contributions of each key component of DFRL-Mol. The experimental results show that the full DFRL-Mol achieved strong performance under both relaxed (δ=0.4) and strict (δ=0.6) structural constraints, and that each module contributed to the final performance. The pre-trained model performed the worst among all variants, but still achieved performance comparable to HierG2G on the QED optimization task, indicating that a certain degree of optimization can be achieved with fitness function guidance alone, without the need for supervised signals. Subsequently, guiding training and reinforcement learning jointly drove the performance improvement of DFRL-Mol. After completing training, the model's performance was already very close to that of the full model. Finally, the combination of curriculum learning and reinforcement learning brought the model to its best performance.

**Fig. S2.** Ablation study. It shows the impact of different models on performance.

**S5 Multi-objective Optimization Process Analysis and Structural Diversity Evaluation**

**S5.1 QED & DRD2 Optimization Convergence Analysis**

**Fig. S3.** QED & DRD2 Optimization Convergence. This figure illustrates the proportion of generated molecules satisfying specific property criteria across optimization steps under structural similarity constraints of δ=0.4 and δ=0.6. Solid lines represent multi-objective optimization tasks, while dashed lines denote single-objective optimization tasks.

Fig. S3 illustrates the changes in the proportions of molecules meeting the property criteria for two properties over 20 optimization steps. The results show that: Under the two similarity constraints, the model exhibited different optimization trajectories. When the constraint was relatively loose (δ=0.4), the explorable chemical space was larger, and the success rates for both DRD2 and QED showed a steady upward trend throughout the optimization process. In the later stage of optimization, the dual-objective success rate gradually approached the single-objective success rate for DRD2, indicating that DRD2 activity was the main bottleneck in the optimization process under this constraint. When the constraint was relatively strict (δ=0.6), the optimization trajectory showed stage-wise characteristics: in the early-to-middle stage (Steps 4-8), DRD2 activity improved first while the QED success rate remained nearly stagnant; in the middle-to-late stage (Steps 12-20), the optimization focus shifted to QED while DRD2 plateaued. Compared with δ=0.4, the gap between the dual-objective and single-objective success rates widened under the strict constraint, indicating that the optimization difficulty increases as the structural constraint tightens.

**S5.2 Property Distribution Analysis**

**Fig. S4.** Property Distribution Evolution. The ridge plots demonstrate the dynamic shifts in the probability densities of individual molecular properties (QED and DRD2) from the initial state to the final optimization phase.

The ridge plots illustrate the changes in molecular properties during multi-objective optimization from the perspective of population distribution. In the early stage of optimization (Steps 0-4), the distributions of all properties shifted to the right. In the subsequent iterations (Steps 4-20), the magnitude of change in the main peak of the distributions decreased, but the overall rightward trend was maintained.

**S5.3 Scaffold Preservation and Diversity Evaluation**

We tracked changes in scaffold retention and diversity during the optimization process under two similarity constraints (δ=0.4 and 0.6), using the following three metrics for quantification:

Lead Similarity (Avg): The average Tanimoto similarity between the generated molecules and the original lead molecules based on Morgan fingerprints, reflecting the degree of shift in the overall chemical space.

Internal Diversity: The complement to 1 of the average similarity between molecules within the set, characterizing the structural richness of the molecular population.

Scaffold Diversity: The proportion of unique scaffolds among all valid molecules, used to quantify diversity at the scaffold level.

The experimental results show that under both constraint conditions, Internal Diversity and Scaffold Diversity remained largely stable during optimization, while Lead Similarity showed a slow declining trend, indicating that the model continuously utilized the allowed exploration space to improve the target properties. Specifically, Internal Diversity remained stable at approximately 0.87, and Scaffold Diversity was maintained at a relatively high level, suggesting that the model preserved high molecular internal diversity and scaffold diversity throughout the optimization process.

**Table S1: Evolution metrics for δ=0.4**

| Step | Lead Similarity (Avg) | Internal Diversity (↑) | Scaffold Diversity (↑) |
| --- | --- | --- | --- |
| Step 4 | 0.500 | 0.874 | 0.99 |
| Step 8 | 0.456 | 0.872 | 0.99 |
| Step 12 | 0.439 | 0.871 | 0.99 |
| Step 16 | 0.436 | 0.869 | 0.99 |
| Step 20 | 0.433 | 0.868 | 0.99 |

**Table S2: Evolution metrics for δ=0.6**

| Step | Lead Similarity (Avg) | Internal Diversity (↑) | Scaffold Diversity (↑) |
| --- | --- | --- | --- |
| Step 4 | 0.633 | 0.874 | 0.97 |
| Step 8 | 0.616 | 0.874 | 0.98 |
| Step 12 | 0.614 | 0.874 | 0.98 |
| Step 16 | 0.610 | 0.873 | 0.98 |
| Step 20 | 0.611 | 0.873 | 0.98 |

**S6 Molecular Docking**

To investigate whether the model, under the guidance of the existing predictors, has learned optimization rules that are relevant to the real world, we employed 3D molecular docking as an independent validation method. We performed docking evaluation by aligning the candidate molecules generated by DFRL-Mol with the crystal structures of the real targets. The results are shown in the table below, where "score" denotes the predicted score based on machine learning methods.

**Table S3: Comparison between predicted oracle scores and DRD2 docking binding affinities**

| smiles | Model_1 | Model_2 | Model_3 | Model_4 | Model_5 | score |
| --- | --- | --- | --- | --- | --- | --- |
| O=C(C1=CC(O)=NC2=CC=CC=C21)CCCCCN1CCN(C2=CC=CC(C(F)(F)F)=C2)CC1 | -11.066 | -10.884 | -10.838 | -10.714 | -10.662 | 0.99 |
| CC1=CC(CCN2CCCC(N3CCN(C4=CC=C(F)C=C4)CC3)CC2)=NO1 | -10.363 | -10.065 | -9.74 | -9.594 | -9.583 | 0.98 |
| N#CC1=CC=CC=C1COC1=CC=CC(N2CCN(CCCCCC3=CC=C(Br)C=N3)CC2)=C1 | -10.352 | -10.28 | -10.106 | -9.921 | -9.894 | 0.99 |
| CC(=O)C1=CC=C(CCCCN2CCN(C3=CC=CC=C3F)CC2)C=C1 | -10.314 | -9.996 | -9.901 | -9.779 | -9.69 | 0.99 |
| O=C(NC1=CN(CCCCCN2CCN(C3=CC=C(C(F)(F)F)C=N3)CC2)N=C1)C1=CC=CS1 | -10.271 | -9.912 | -9.64 | -9.638 | -9.578 | 0.87 |
| O=C(NCCCCN1CCC2=CC=CC=C2C1)C1=CC=CC(F)=C1Cl | -10.225 | -10.138 | -10.074 | -9.918 | -9.789 | 0.91 |
| CCCN(CCNCC1=CC=C(OC(F)(F)F)C=C1)C1COC2=CC=CC=C2C1 | -10.108 | -9.95 | -9.499 | -9.495 | -9.464 | 0.98 |
| CC(NCCCCN1CCOC(C2=CC=CC=C2)C1)C1=CC=C(F)C=C1 | -9.907 | -9.854 | -9.688 | -9.571 | -9.471 | 0.94 |
| O=C(NC(CC1=CC=CC=C1)NCCCN1CCN(C2=NC=CC=N2)CC1)C1=CC=C(Cl)C=C1 | -9.858 | -9.841 | -9.694 | -9.688 | -9.619 | 0.92 |
| COC1=CC=CC=C1N1CCN(CCCCNC(=O)N=CC2=CC=C(Cl)C=C2Cl)CC1 | -9.75 | -9.284 | -9.15 | -9.092 | -8.886 | 0.99 |
| O=S(=O)(C1=CC=CC=C1)NCCCCN1CCN(C2=CC=CC(Cl)=C2)CC1 | -9.66 | -9.149 | -8.928 | -8.761 | -8.679 | 0.96 |
| O=C(CCCCN1CCN(C2=CC=CC=C2)CC1)C1=CC=CC=C1 | -9.658 | -9.579 | -9.45 | -9.374 | -9.137 | 0.99 |
| CCC1(C2=CC=CC=C2)CCN(CC2=CC=C(Cl)C=C2)CC1 | -9.357 | -8.728 | -8.674 | -8.604 | -8.595 | 0.94 |
| CC1=CC=C(NC(C)CCCN2CCN(C3=NC=CC=C3F)CC2)C=C1 | -9.327 | -9.29 | -9.284 | -9.249 | -8.949 | 0.97 |
| COC1=CC=C(N2CCN(CCCOC3=CC(C)=C(Cl)C=C3C)CC2)C=C1 | -9.183 | -9.052 | -9.039 | -8.925 | -8.883 | 0.97 |
| O=C(CCCCN1CCCC1)N1CCC(C2=NC3=CC=CC=C3S2)CC1 | -8.961 | -8.572 | -8.244 | -8.169 | -7.99 | 0.97 |
| O=C(C1=CC=CC(F)=C1)N1CCCC(CCCN2CCCCC2)C1 | -8.879 | -8.819 | -8.584 | -8.396 | -8.378 | 0.87 |
| C#CCCCN1CCN(C2=COC(C3=CC=CC(Cl)=C3)=N2)CC1 | -8.837 | -8.549 | -8.128 | -8.045 | -7.996 | 0.97 |
| COC1=CC=CC=C1CCCN1CCN(C2=NC=CC=N2)CC1 | -8.38 | -8.365 | -8.069 | -7.723 | -7.557 | 0.98 |
| CCCN1CCCCC(CC2CSC3=CC=CC=C32)C1 | -6.917 | -6.882 | -6.842 | -6.781 | -6.632 | 0.93 |

**Table S4: Comparison between predicted oracle scores and JNK3 docking binding affinities.**

| smiles | Model_1 | Model_2 | Model_3 | Model_4 | Model_5 | score |
| --- | --- | --- | --- | --- | --- | --- |
| C1=CC(Cl)=CC=C1NC2=NC=CC(C3=CC(C(=O)N)=CC(N4CCOCC4)=C3)=N2 | -9.131 | -9.045 | -8.941 | -8.907 | -8.877 | 0.74 |
| C1=CC(C)=CC=C1NC2=NC=CC(C3=CC=C(N4CCC(O)CC4)C=C3)=N2 | -9.073 | -8.556 | -8.524 | -8.193 |  | 0.72 |
| C1=CC(Cl)=CC=C1NC2=NC=CC(C3=CC(F)=CC(N4CCOCC4)=C3)=N2 | -8.972 | -8.918 | -8.75 | -8.721 | -8.699 | 0.78 |
| C1=CC(C)=CC=C1NC2=NC=CC(C3=CC=C(N4CCOCC4)N=C3)=N2 | -8.967 | -8.846 | -8.335 | -8.33 | -8.323 | 0.69 |
| C1=CC=CC=C1NC2=NC=C(Cl)C(NC3=CC=CC=C3C(N)=O)=N2 | -8.952 | -8.548 | -8.499 | -8.416 | -8.289 | 0.94 |
| C1=CC(C)=CC=C1NC2=NC=CC(C3=CC=C(N4CCCCC4)C=C3)=N2 | -8.908 | -8.761 | -8.543 | -8.502 | -8.439 | 0.72 |
| C1=CC(C2CC2)=CC=C1NC2=NC=CC(C3=CC=CC=C3)=N2 | -8.778 | -8.758 | -8.7 | -8.577 | -8.382 | 0.72 |
| C1=CC(F)=CC=C1NC2=NC=CC(C3=CC=CC=C3)=N2 | -8.771 | -8.603 | -8.427 | -8.386 | -8.272 | 0.72 |
| C1=CC=CC=C1NC2=NC=CC(C3=CC=CC=C3)=N2 | -8.71 | -8.671 | -8.387 | -8.233 | -8.191 | 0.7 |
| C1=CC(Cl)=CC=C1NC2=NC=CC(C3=CC(C#N)=CC(N4CCOCC4)=C3)=N2 | -8.633 | -8.496 | -8.431 | -8.397 | -8.332 | 0.79 |
| C1=CC(C)=CC=C1NC2=NC=CC(C3=CC=C(Cl)C=C3)=N2 | -8.595 | -8.321 | -8.303 | -8.25 | -8.111 | 0.71 |
| C1=CC(Cl)=CC=C1NC2=NC=CC(C3=CC=CC=C3)=N2 | -8.583 | -8.574 | -8.434 | -8.388 | -8.387 | 0.74 |
| C1=CC(N2CCOCC2)=CC=C1NC2=NC=CC(C3=CC=CC=C3)=N2 | -8.512 | -8.402 | -8.259 | -8.25 |  | 0.82 |
| C1=CC(Cl)=CC=C1NC2=NC=CC(C3=CC=CC(N4CC4)=C3)=N2 | -8.469 | -8.256 | -8.202 | -8.104 | -8.081 | 0.72 |
| C1=CC(N2CCCC2)=CC=C1NC2=NC=CC(C3=CC=CC=C3)=N2 | -8.462 | -8.436 | -8.4 | -8.101 | -8.076 | 0.73 |
| C1=CC(N)=CC=C1NC2=NC=CC(C3=CC=CC=C3)=N2 | -8.425 | -8.229 | -8.192 | -8.026 | -8.004 | 0.75 |
| C1=CC(N)=CC=C1NC2=NC=CC(C3=CC=C(Cl)C=C3)=N2 | -8.359 | -8.11 | -7.986 | -7.723 | -7.721 | 0.74 |
| C1=CC(C#N)=CC=C1NC2=NC=CC(C3=CC=CC(C#N)=C3)=N2 | -8.141 | -7.996 | -7.872 | -7.797 | -7.624 | 0.71 |
| C1=CC(C#N)=CC=C1NC2=NC=CC(C3=CC=C(Cl)C=C3)=N2 | -8.087 | -8.05 | -7.978 | -7.821 | -7.73 | 0.76 |
| C1=CC(C#N)=CC=C1NC2=NC=CC(C3=CC=CC(N4CC4)=C3)=N2 | -8.066 | -8.052 | -7.981 | -7.796 | -7.719 | 0.77 |

**Table S5: Comparison between predicted oracle scores and GSK3b docking binding affinities.**

| smiles | Model_1 | Model_2 | Model_3 | Model_4 | Model_5 | score |
| --- | --- | --- | --- | --- | --- | --- |
| C1=CC(Cl)=CC=C1NC2=NC=CC(C3=CC=NC(NC(=O)C4CCCC4)=C3)=N2 | -10.3 | -9.767 | -9.728 | -9.652 | -9.647 | 0.83 |
| C1=CC(Cl)=CC=C1NC2=NC=CC(C3=CC=NC(NC4CCCCC4)=N3)=N2 | -10.026 | -9.226 | -8.933 | -8.923 | -8.84 | 0.84 |
| C1=CC(C)=CC=C1NC2=NC=CC(C3=CC=NC(NC4CCCC4)=N3)=N2 | -9.97 | -9.837 | -9.252 | -8.98 | -8.816 | 0.85 |
| C1=CC(Cl)=CC=C1NC2=NC=CC(C3=CC(F)=CC(N4CCOCC4)=C3)=N2 | -9.893 | -9.482 | -9.374 | -9.347 | -9.091 | 0.83 |
| C1=CC(C)=CC=C1NC2=NC=CC(C3=CC=C(N4CCCC4)C=C3)=N2 | -9.82 | -9.42 | -9.367 | -8.874 | -8.809 | 0.83 |
| C1=CC(Cl)=CC=C1NC2=NC=CC(C3=CC(C#N)=CC(N4CCOCC4)=C3)=N2 | -9.815 | -9.507 | -9.423 | -9.367 | -9.173 | 0.83 |
| C1=CC(Cl)=CC=C1NC2=NC=CC(C3=CC=NC(NC4CCCC4)=N3)=N2 | -9.704 | -8.947 | -8.73 | -8.71 | -8.564 | 0.85 |
| C1=CC(Cl)=CC=C1NC2=NC=CC(C3=CC=NC(NC4CCOCC4)=N3)=N2 | -9.527 | -9.297 | -8.721 | -8.656 | -8.648 | 0.84 |
| C1=CC(Cl)=CC=C1NC2=NC=CC(C3=CC=C(N4CCCCC4)C=C3)=N2 | -9.428 | -9.283 | -9.194 | -9.17 | -9.02 | 0.86 |
| C1=CC(C)=C(Cl)C=C1NC2=NC=CC(C3=CC=NC(NC4CC4)=C3)=N2 | -9.29 | -9.263 | -9.181 | -9.105 | -9.066 | 0.85 |
| C1=CC=CC=C1NC2=NC=C(Cl)C(NC3=CC=CC=C3C(N)=O)=N2 | -9.289 | -9.17 | -9.126 | -8.94 | -8.741 | 0.85 |
| C1=CC(C#N)=CC=C1NC2=NC=CC(C3=CC=C(Cl)C=C3)=N2 | -9.239 | -9.218 | -9.108 | -9.082 | -8.875 | 0.85 |
| C1=CC(Cl)=CC=C1NC2=NC=CC(C3=CC(F)=CC(F)=C3)=N2 | -9.151 | -9.121 | -9.003 | -8.985 | -8.772 | 0.88 |
| C1=CC(Cl)=CC=C1NC2=NC=CC(C3=CC=CC(C#N)=C3)=N2 | -9.099 | -8.731 | -8.531 | -8.509 | -8.508 | 0.83 |
| C1=CC(Cl)=C(Cl)C=C1NC2=NC=CC(C3=CC=NC(NC4CC4)=C3)=N2 | -9.08 | -9.072 | -8.939 | -8.822 | -8.533 | 0.86 |
| C1=CC(Cl)=CC=C1NC2=NC=CC(C3=CC=CC(Cl)=C3)=N2 | -9.018 | -8.994 | -8.77 | -8.692 | -8.666 | 0.83 |
| C1=CC=C(Cl)C=C1NC2=NC=CC(C3=CC=CC=C3)=N2 | -8.997 | -8.82 | -8.783 | -8.641 | -8.571 | 0.93 |
| C1=CC(Cl)=CC=C1NC2=NC=CC(C3=CC=CC(OC)=C3)=N2 | -8.874 | -8.674 | -8.537 | -8.458 | -8.379 | 0.84 |
| C1=CC=CC=C1NC2=NC=CC(C3=CC=CC=C3)=N2 | -8.772 | -8.652 | -8.527 | -8.474 | -8.35 | 0.87 |
| C1=CC(Cl)=CC=C1NC2=NC=CC(C3=CC=NC(NC4CC4)=N3)=N2 | -8.771 | -8.698 | -8.628 | -8.554 | -8.521 | 0.87 |

**S7 Case Studies**

To conduct an in-depth analysis of the model's behavior across different optimization scenarios, we selected representative cases for QED, logP, and DRD2 binding affinity, and analyzed them from the perspectives of group properties and mechanisms of action:

**S7.1 QED Optimization**

**Fig. S5.** Case study of QED optimization. The left figure shows the molecular structures before and after optimization. The right figure shows the change in properties.

QED quantifies drug-likeness by evaluating the alignment of molecular properties with the drug-like chemical space, and is correlated with multiple molecular properties. As shown in Fig. S5 (left: molecular structures before and after optimization; right: radar plot of property changes), in this case, DFRL-Mol replaced the terminal iodine atom (-I) of the lead molecule with an amide group (-CONH_2_), increasing the QED score from 0.41 to 0.85.

The lead molecule consists of a polycyclic scaffold with a terminal iodine atom. It exhibits high lipophilicity (logP=5.69), lacks a hydrogen bond network (TPSA=0, both hydrogen bond acceptors and donors are 0), and is rigid due to its fused polycyclic structure (number of rotatable bonds is 0). These factors together contribute to its low drug-likeness. While preserving the core scaffold, DFRL-Mol replaced -I with -CONH₂, achieving drug-likeness optimization from two aspects: first, introducing a polar surface (TPSA increased to 43.09 Å²) and a hydrogen bond network (both acceptors and donors increased from 0 to 2), thereby reducing the excessive lipophilicity; second, increasing structural flexibility (rotatable bonds increased from 0 to 1), providing the molecule with conformational adaptability required for target binding. This case demonstrates that DFRL-Mol can achieve multi-faceted property improvement through rational functional group editing while preserving the scaffold.

**S7.2 LogP Optimization**

**Fig. S6.** Case study of logP optimization. The figure shows the change in logP before and after optimization.

LogP is a commonly used metric for measuring molecular lipophilicity, which affects drug absorption and membrane permeability. In the case shown, DFRL-Mol replaced a polar nitrogen atom on the heterocyclic ring with a carbon atom bearing an ethyl side chain, changing the molecule's logP from 0.44 to 1.60. The lead molecule contains multiple heteroatoms, has a high polar surface area, and exhibits low lipophilicity. While preserving the scaffold, the model removed the polar nitrogen atom and introduced a hydrophobic ethyl group, thereby increasing the molecule's lipophilicity.

**S7.3 DRD2** **Optimization**

**Fig. S7.** Case study of DRD2 optimization. The figure shows the binding of the small molecule to the target before and after optimization.

To evaluate the binding ability of the molecules generated by DFRL-Mol to the target, we performed molecular docking experiments.

The lead molecule contains an isoxazole and a fluorophenyl scaffold. The model performed a decarbonylation modification, reducing the amide bond (-C(=O)N-) to an aliphatic amine (-CH_2_-N-), which increased the number of rotatable bonds from 3 to 5. As shown in Fig. S7, this structural change provided the molecule with more conformational sampling space in the receptor pocket, and the docking conformation showed improved complementarity with the pocket. The docking results show that the binding energy of the optimized molecule changed from -8.617 kcal/mol to -10.363 kcal/mol.

**S7.4 Literature-supported Case Study**

Established medicinal chemistry strategies provide a benchmark for evaluating the interpretability of molecular optimization models. The discovery of the antidepressant Agomelatine serves as a classic example of lipophilicity-driven optimization. In this historical case, researchers optimized the endogenous hormone Melatonin by replacing its electron-rich indole scaffold with a naphthalene scaffold. This bioisosteric substitution (N to C) was explicitly designed to increase lipophilicity, thereby enhancing the drug's penetration across the blood-brain barrier.

Fig. S8 illustrates the correspondence between this established medicinal chemistry rule and the strategies learned by our model. Fig. S8 a depicts the historical optimization from Melatonin to Agomelatine, where the removal of the polar nitrogen atom resulted in an increase in logP. Correspondingly, Fig. S8 b-d display analogous optimization trajectories generated by DFRL-Mol. These examples demonstrate that the model has successfully captured expert-like chemical intuition from the data. By performing similar N to C substitutions on diverse substrates (Fig. S8 b-d), DFRL-Mol effectively modulates physicochemical properties.

**Fig. S8.** Alignment of DFRL-Mol’s policy with the Agomelatine precedent. (a) Historical N to C bioisosteric replacement (Melatonin to Agomelatine) to increase lipophilicity. (b-d) Analogous optimization steps generated by DFRL-Mol.

**S7.5 Failure Case Analysis**

**Fig. S9.** A failure case in QED & DRD2 optimization. The left figure shows the molecular structures before and after optimization. The right figure shows the change in properties.

Structural comparison shows that the model performed N-alkylation of the amine while the scaffold remained unchanged. After the primary amine was converted to a tertiary amine, the number of hydrogen bond donors (H-Donors) decreased from 3 to 1, the topological polar surface area (TPSA) decreased from 57.9 to 35.2, logP increased from 5.84 to 6.83, and the number of rotatable bonds increased from 4 to 6. These changes are consistent with the observed trends of increased DRD2 activity and decreased QED, reflecting the trade-off between the two objectives during the optimization process.

**S8 Success Rate Analysis of Transformation Patterns**

We calculated the success rates and average property improvements for representative transformation patterns. This experiment was conducted under unconstrained conditions with only single-step optimization, to exclude the influence of structural similarity constraints and the fitness function. We collected all valid transformation cases from the logP optimization tasks and excluded those with fewer than 20 samples to ensure the representativeness of the results. The results are shown in Tables S6 and S7: after reinforcement learning, the model showed improved success rates and average improvements on most transformation patterns. This result indicates that the model is able to learn structural transformation patterns with higher success rates.

**Table S6: Comparison of success rates and score improvements for different group replacements after CDSPO.**

| Source_Category | Target_Category | Success_Rate | Avg_Improv |
| --- | --- | --- | --- |
| Nitrogen_Group | Halogen | 100.00% | 1.63 |
| Unsaturation | Halogen | 100.00% | 1.55 |
| Oxygen_Group | Halogen | 100.00% | 1.47 |
| Nitrogen_Group | Nitrogen_Group | 100.00% | 0.93 |
| Unsaturation | Carbon_Chain | 99.48% | 1.53 |
| Carbon_Chain | Carbon_Chain | 99.32% | 0.88 |
| Nitrogen_Group | Carbon_Chain | 99.01% | 1.55 |
| Oxygen_Group | Carbon_Chain | 98.36% | 1.20 |
| Carbon_Chain | Halogen | 97.44% | 0.92 |
| Oxygen_Group | Oxygen_Group | 97.06% | 0.74 |
| Halogen | Halogen | 95.24% | 0.69 |
| Unsaturation | Unsaturation | 94.12% | 1.23 |
| Oxygen_Group | Unsaturation | 92.86% | 0.88 |
| Carbon_Chain | Unsaturation | 88.89% | 0.70 |
| Nitrogen_Group | Unsaturation | 82.14% | 0.86 |

**Table S7: Comparison of success rates and score improvements for different group replacements before CDSPO.**

| Source_Category | Target_Category | Success_Rate | Avg_Improv |
| --- | --- | --- | --- |
| Nitrogen_Group | Unsaturation | 90.98% | 0.74 |
| Nitrogen_Group | Carbon_Chain | 89.19% | 0.55 |
| Oxygen_Group | Unsaturation | 81.82% | 0.53 |
| Carbon_Chain | Carbon_Chain | 73.08% | 0.18 |
| Unsaturation | Halogen | 70.27% | 0.37 |
| Nitrogen_Group | Nitrogen_Group | 70.00% | 0.31 |
| Carbon_Chain | Unsaturation | 63.46% | 0.08 |
| Carbon_Chain | Halogen | 57.69% | 0.08 |
| Unsaturation | Unsaturation | 57.21% | 0.05 |
| Unsaturation | Carbon_Chain | 46.34% | 0.00 |
| Halogen | Unsaturation | 36.84% | -0.26 |
| Carbon_Chain | Nitrogen_Group | 18.75% | -0.47 |
| Unsaturation | Oxygen_Group | 9.68% | -0.75 |
| Unsaturation | Nitrogen_Group | 9.38% | -0.52 |

**S9 MO Molecule Generation**

**S9.1 Detailed Results**

**Table S8: MO Molecule Generation Results**

| Properties | APS | Nov | Validity | Uniqueness | Scaffold Diversity | Internal Diversity |
| --- | --- | --- | --- | --- | --- | --- |
| GSK3β & JNK3 | 0.841 | 100% | 100% | 100% | 39.7% | 0.4399 |
| GSK3β & JNK3 & QED & SA | 0.804 | 100% | 100% | 100% | 41.4% | 0.5638 |

The complete results of the generation tasks are shown in Table S8, where:

**Validity:** The proportion of molecules that can be successfully parsed as valid chemical structures.

**Uniqueness:** A measure of whether SMILES strings appear repeatedly.

**Internal Diversity:** This metric is quantified by calculating the average Tanimoto distance (based on Morgan fingerprints with radius 2 and 2048 bits) across all pairs of molecules in the set of generated unique molecules.

**Scaffold Diversity:** The Murcko scaffolds of the generated molecules are extracted, and the ratio of the number of unique scaffolds to the total number of molecules is calculated.

In both the dual-objective and four-objective tasks, the model achieved 100% on novelty, validity, and uniqueness. This indicates that the model is capable of exploring chemical space with entirely new, valid, and non-repetitive molecules. In both tasks, scaffold diversity and internal diversity were relatively low, showing a certain degree of concentration. This result is jointly caused by two reasons. First, DFRL-Mol adopts an iterative modification approach, which leads to scaffold similarity. Second, highly active molecules tend to share conserved core pharmacophores.

**S9.2 Single-Property Distributions in MO Molecule Generation**

In the four-objective generation task, the distribution of each property is shown in Fig. S10:

**Fig. S10.** Property distributions in the four-objective optimization task.

In the dual-objective generation task, the distribution of each property is shown in Fig. S11:

**Fig. S11.** Property distributions in the two-objective optimization task.

**S10 Overlap Analysis of Training and Test Sets**

We analyzed the overlap between the benchmark test set and the datasets used in each training stage. The analysis included exact matching of canonical SMILES, matching after desalting, scaffold matching, and Tanimoto similarity matching. Since only one-dimensional SMILES were used, stereochemical information was not included. Molecules that were completely duplicated had already been removed during the preliminary data processing stage. For paired supervised datasets, both ends of each molecular pair were separately compared against the test set. The results of the analysis are shown in Table S9.

**Table S9: Overlap analysis of training and benchmark sets.**

**A. Pre-training Set**

| Ratio(%) | Exact | Desalted | Scaffold | Tanimoto ≥ 0.85 |
| --- | --- | --- | --- | --- |
| QED | 0 | 0 | 18.75 | 0 |
| logP | 0 | 0 | 19.38 | 0 |
| DRD2 | 0 | 0 | 16.70 | 0.40 |
| QED&DRD2 | 0 | 0 | 17.25 | 1.62 |

**B. Fine-tuning**

| Ratio(%) | Exact | Desalted | Scaffold | Tanimoto ≥ 0.85 |
| --- | --- | --- | --- | --- |
| QED | 0 | 0.12 | 33.12 | 0.38 |
| logP | 0 | 0 | 30.63 | 0 |
| DRD2 | 0 | 0 | 34.50 | 0.30 |
| QED&DRD2 | 0 | 0 | 25.62 | 1 |

**C. CDSPO**

| Ratio(%) | Exact | Desalted | Scaffold | Tanimoto ≥ 0.85 |
| --- | --- | --- | --- | --- |
| QED | 0 | 0 | 36.88 | 0 |
| logP | 0 | 0 | 31.62 | 0.12 |
| DRD2 | 0 | 0 | 31.80 | 0.20 |
| QED&DRD2 | 0 | 0 | 36.00 | 2.62 |

We removed from the test set all molecules that overlapped with the training set (covering all metrics in the table), constructed a non-overlapping test set, and re-ran the benchmark experiments on this test set. The results are shown in Table S12. The results show that, under the stricter test set conditions, the model's performance on all metrics showed only minor fluctuations compared with the original test set. Accordingly, the risk of data leakage is largely ruled out.

**Fig. S12.** Performance Comparison of Original and Scaffold-filtered Test Sets

**S11 ADMET-Targeted Multi-Objective Molecular Generation**

We incorporated representative ADMET and toxicity endpoints into the reward function and evaluation protocol. Following the task construction scheme of "target activity + QED/SA + ADMET safety," we designed and added three new multi-objective optimization tasks in the revised manuscript:

Task 1: DRD2 activity + QED + hERG (cardiotoxicity) + Oral Bioavailability (Bioavailability_Ma), with cardiovascular safety and oral administration as constraints.

Task 2: GSK3β activity + SA + DILI (hepatotoxicity) + CYP3A4 (enzyme inhibition), with hepatotoxicity and drug-drug interaction as constraints.

Task 3: JNK3 activity + QED + Half-Life (metabolic stability, Half_Life_Obach), with metabolic stability as the constraint.

To control for differences in scale across different ADMET property predictions, we standardized the raw outputs of each predictor. All properties were uniformly mapped to the [0, 1] interval, with larger values indicating more favorable outcomes in that dimension. The specific mapping rules are as follows:

**Oral bioavailability:** The predictor outputs a probability value that already falls within the [0, 1] interval; this value was used directly as the reward signal.

**Toxicity and inhibition properties** (hERG, DILI, CYP3A4): For these properties, smaller raw values indicate higher safety. To align the direction, we applied the transformation f(x)=1 − x to map them to positive scores.

**Half-life:** Values that are too short or too long are both undesirable for drug metabolism. We therefore applied a piecewise mapping strategy: values between 2 and 24 hours were mapped to 1.0; values between 0 and 2 hours were mapped to x / 2; values between 24 and 60 hours were mapped to (60 − x) / 36.0; all other values were mapped to 0.0.

The experimental results are shown in Table S10. The results demonstrate that DFRL-Mol can effectively handle multi-objective generation tasks with incorporated ADMET metrics. In Tasks 2 and 3, it achieved relatively high APS values. In Task 1, the APS was lower than in the other two tasks, indicating more severe property conflicts within this task.

Finally, the diversity metrics reflect the model's adaptive exploration strategy under different constraints. In Task 2, scaffold diversity was relatively low while internal diversity remained stable, suggesting that the model tended to perform side-chain optimization on a limited set of safe scaffolds (internal diversity still remained at 54.6%). In contrast, in Task 3, which includes only three subtasks, scaffold diversity reached 80.1%, demonstrating that the model is capable of broader exploration when physicochemical constraints allow.

**Table S10: MO Molecule generation result with ADMET**

| Properties | APS | Nov | Validity | Uniqueness | Scaffold Diversity | Internal Diversity |
| --- | --- | --- | --- | --- | --- | --- |
| DRD2 & QED & hERG & Bioavailability_Ma | 67.1% | 100% | 100% | 100% | 38.0% | 66.6% |
| GSK3β & SA & DILI & CYP3A4 | 83.3% | 100% | 100% | 100% | 26.0% | 54.6% |
| JNK3 & QED & Half_Life_Obach | 84.0% | 100% | 100% | 100% | 80.1% | 56.9% |

Fig. S13 presents three representative molecules.

**Fig. S13.** ADMET-targeted multi-objective molecular generation example
